# Microstates-based resting frontal alpha asymmetry approach for understanding affect and approach/withdrawal behavior

**DOI:** 10.1038/s41598-020-61119-7

**Published:** 2020-03-06

**Authors:** Ardaman Kaur, Vijayakumar Chinnadurai, Rishu Chaujar

**Affiliations:** 1 NMR Research Centre, Institute of Nuclear Medicine and Allied Sciences, Lucknow Road, Timarpur, Delhi, 110054 India; 2 Department of Applied Physics, Delhi Technological University, Shahbad Daulatpur, Main Bawana Road, Delhi, 110042 India

**Keywords:** Neuroscience, Cognitive neuroscience

## Abstract

The role of resting frontal alpha-asymmetry in explaining neural-mechanisms of affect and approach/withdrawal behavior is still debatable. The present study explores the ability of the quasi-stable resting EEG asymmetry information and the associated neurovascular synchronization/desynchronization in bringing more insight into the understanding of neural-mechanisms of affect and approach/withdrawal behavior. For this purpose, a novel frontal alpha-asymmetry based on microstates, that assess quasi-stable EEG scalp topography information, is proposed and compared against standard frontal-asymmetry. Both proposed and standard frontal alpha-asymmetries were estimated from thirty-nine healthy volunteers resting-EEG simultaneously acquired with resting-fMRI. Further, neurovascular mechanisms of these asymmetry measures were estimated through EEG-informed fMRI. Subsequently, the Hemodynamic Lateralization Index (HLI) of the neural-underpinnings of both asymmetry measures was assessed. Finally, the robust correlation of both asymmetry-measures and their HLI’s with PANAS, BIS/BAS was carried out. The standard resting frontal-asymmetry and its HLI yielded no significant correlation with any psychological-measures. However, the microstate resting frontal-asymmetry correlated significantly with negative affect and its neural underpinning’s HLI significantly correlated with Positive/Negative affect and BIS/BAS measures. Finally, alpha-BOLD desynchronization was observed in neural-underpinning whose HLI correlated significantly with negative affect and BIS. Hence, the proposed resting microstate-frontal asymmetry better assesses the neural-mechanisms of affect, approach/withdrawal behavior.

## Introduction

Understanding the neural mechanisms associated with functional hemispheric asymmetry of affect, approach/withdrawal measures is one of the core focuses in neuroscience. Numerous studies revealed an association of functional hemispheric asymmetry with positive/negative affect and approach/withdrawal dichotomy. This linkage was initially observed in many studies where left hemispheric lesion affected the perception of positive emotions whilst damage to the right hemisphere impaired the perception of negative emotions^1–3^. Subsequently, there was a surge in elucidating the role of frontal hemispheric asymmetry based on the alpha signature of electroencephalography (EEG) in manifesting the individual differences in affect and approach/withdrawal measures^4–6^. Davidson *et al*.^7–9^, in their studies, suggested the lateralization of the prefrontal cortex (PFC) with respect to positive/motivational valence. Thus, the right PFC was observed to be linked with avoidance/negative emotion and left PFC with approach/positive emotion. Nevertheless, Carver and Harmon-Jones^10^ showed the association of left hemisphere with negative emotion anger and thus proposed to eliminate the differentiation of positive and negative valence from the affective model. Subsequently, a larger number of studies concentrated on EEG frontal asymmetry through the induction of emotional/motivational states or tasks to understand the neural mechanisms associated with the evoked approach/withdrawal behavior^11–18^ and other specific tasks^19^. This has led to ample literature which examined alterations in frontal EEG asymmetry in clinical and healthy populations^20–28^.

Although the aforementioned studies have proved EEG based frontal asymmetry assessment as a reliable indicator of affect, approach/withdrawal behavior during emotional tasks, it’s validity in healthy individuals during resting still remains ambiguous. In one large resting EEG study, Tomarken *et al*.^29,30^ revealed a significant negative correlation of resting Frontal asymmetry (FA; channel pair: F4, F3) with negative affect and positive correlation of resting Anterior Temporal Asymmetry (ATA; channel pair: T4, T3) with positive affect for female subjects. Jacobs and Snyder^31^, in their study, revealed the negative correlation of resting Frontal Temporal Asymmetry (FTA; channel pair: F8, F7) with negative affect in men, further Hall and Petruzzello^32^ showed that resting FA positively predicted the positive affect of both sexes. Pertaining to approach and withdrawal measures, studies by Harmon-Jones and Allen^33^ and De Pascalis *et al*.^34^ reported a significant positive correlation of approach measure, Behavioral Activation System (BAS) with resting FA. The aforementioned studies are in sync with the hypothesis that positive affect correlates positively with alpha asymmetry $(\mathrm{ln}\,(\alpha lph{a}^{Right})-\,\mathrm{ln}\,(\alpha lph{a}^{Left}))$ and links to the left hemisphere, howbeit negative affect correlates negatively with the same and associates with the right hemisphere. Conversely, in another study^35^, absolutely no significant relationship was observed between resting FA and measures of positive and negative valence for both sexes. Similarly, Schneider *et al*.^36^ observed an absence of correlation between resting alpha FA and measures of approach/withdrawal behavior. In contradiction to the above hypothesis, Hagemann *et al*.^37^ showed that subjects exhibiting greater relative left-hemispheric resting cortical activation at the anterior temporal site reported more intense NA in response to negative stimuli. Further, in the same line of research^38^, it was found that subjects scoring high on NA, demonstrated greater relative left-sided resting cortical activation at the anterior temporal region than subjects scoring low on NA.

Most findings of the aforementioned literature are based on two fundamental assumptions. Firstly, the above studies assume the acquired EEG to possess only stable cognitive information. Hence, these studies correlate the single session EEG information directly with affect and approach/ withdrawal measures. However, many studies^29,39^ revealed that the stable EEG patterns across previous sessions showed the interrelation of affect and approach/ withdrawal measures with frontal alpha asymmetry. This brings the importance of assessing the stable EEG patterns and information from single session recordings as unstable EEG information may be influenced by interference from many cognitive factors. Recent EEG studies of wakeful rest have shown that global electrical brain activity on scalp remains semi-stable for transient periods^40,41^. In specifics, there exists a finite number of scalp potential topographies in spontaneous resting EEG activity that remains stable for a definite time before rapidly shifting to a different topography that once again attains a stable state. These distinct epochs of topographic stability have been referred to as ‘EEG microstates’. Lehman *et al*.^42^ substantiated that EEG microstates represent blocks of consciousness, and these microstates are modulated by the content of the thoughts. Additionally, Milz *et al*.^43^ postulated the role of intracranial sources in the alpha band in predominantly determining these EEG microstate topographies. Further, Shafi *et al*.^44^, in their study, highlighted the role of microstates in individual variability of human fluid intelligence and in response to cognitive training. Howbeit, there is no study to date that has explored the quasi-stable state as assessed by EEG microstates for understanding frontal hemispheric asymmetry. Also, their ability over standard EEG frontal asymmetry in explaining affect and approach/withdrawal dichotomy is still unmapped.

Further, the second important assumption is that EEG alpha power is inversely^45–47^ related to neural activation. Hence, an increase in neural activation in the left hemisphere is associated with the increase observed in frontal asymmetry scores. This enables us in concluding that the positive correlation of affect and approach/withdrawal measures with frontal asymmetry score $(\mathrm{ln}\,(\alpha lph{a}^{Right})-\,\mathrm{ln}\,(\alpha lph{a}^{Left}))$ is the resultant of left hemispherical neuronal activity and vice versa. However, recently, many neuro-vascular studies^48–51^ have observed alpha-BOLD synchronization wherein the alpha power correlates positively with neural activation during task engagement. Hence, there is a need to fully understand the neurovascular coupling and neural underpinning associated with frontal EEG asymmetry^5^ and how alpha-BOLD synchronization or desynchronization during resting-state associates with affect and approach/withdrawal behavior. Few researchers brought better understanding by studying the role of hemispheric asymmetry in affect, approach/avoidance behavior through functional MR imaging. Rohr *et al*.^52^ concluded that the affective elements in the underlying organization of emotion are predominantly associated with the network of right-hemispheric regions. Lindquist *et al*.^53^ proposed that the implementation of valence depends on a set of valence-general limbic and paralimbic brain regions. Though the above studies gave significant insights, the congruence between resting-EEG frontal alpha asymmetry and resting-fMRI is still uncharted. This is vital for a better understanding of neuro-vascular aspects of resting frontal asymmetry and their association with affect and approach/withdrawal behavior.

Hence, the present study proposes an EEG microstate based approach for assessment of quasi-stable frontal hemispherical asymmetry measures of resting-state affect and approach/withdrawal behavior. It further aims to compare the performance of microstate based frontal hemispheric asymmetry against the standard resting EEG frontal asymmetry measures. For this purpose, resting EEG was acquired from a sample of 39 healthy male subjects. This multichannel resting-EEG signal from all subjects was parsed into a limited number of distinct quasi-stable microstates. These microstates were back-fitted to each subject’s EEG data to obtain microstate time-series data specific to each subject. The microstate time-series was further filtered at alpha frequency band and EEG microstate based frontal asymmetry measures were derived from channel pairs F4/F3 (FA) and F8/F7 (FTA). Further, the robust correlation of both standard and EEG microstate based frontal hemispheric asymmetry with positive/negative affect (PANAS) and approach (BAS)/withdrawal (BIS) behavior was carried out.

Moreover, the study focuses on bringing a better understanding of neural mechanisms associated with functional hemispheric asymmetry of affect and approach/ withdrawal behavior during resting-state. For this purpose, standard and microstates based resting EEG frontal asymmetries were subjected to the EEG informed fMRI approach and the associated neural underpinning of both EEG frontal asymmetries were independently estimated. Thereafter, the hemodynamic lateralization index (HLI) based on the amplitude of hemodynamic response function (HRF) of regions part of the neural underpinning of both EEG frontal asymmetries were assessed. Further, the estimated HLI was subjected to a robust correlation with resting-state affect and approach/ withdrawal psychological scores. Finally, the results were analyzed to understand the ability of proposed EEG microstate estimates in revealing neural-vascular insights of association of functional hemispherical asymmetry with resting-state affect and approach/ withdrawal behavior.

## Results

**Figure 1:**
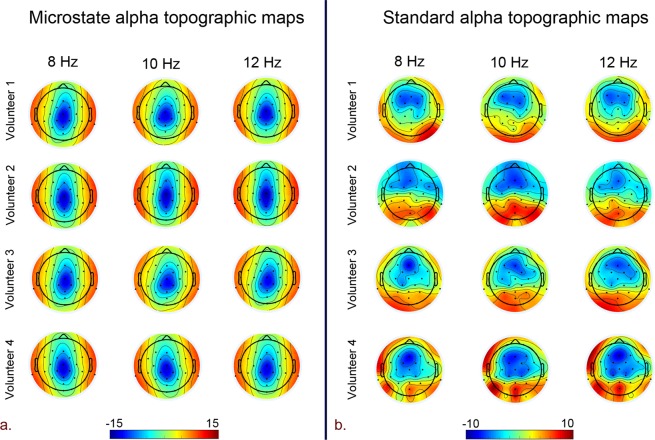
Topographic EEG maps of spectral power density for the alpha band for. (**a**) Proposed microstate based analysis and. (**b**) Standard analysis (CSD referenced). The color bar represents the log-transformed spectral power density (10*log10 (µv^2^/Hz)) where red represents the maximum and blue represents the minimum values.

Our study focused on exploring the ability of quasi-stable EEG microstate based frontal alpha hemispherical asymmetry measures against standard EEG frontal alpha asymmetry measures in explaining the resting state affect and approach/ withdrawal behavior for healthy young male volunteers during 1-time measurement. The standard alpha topographic maps (CSD referenced) and microstate alpha topographic maps are shown in Fig. 1. Evidently, the maps of standard alpha topography (CSD referenced) in Fig. 1b reveal the typical parietal-occipital alpha activity for eyes-closed resting-state condition^54,55^. However, the parietal-alpha activity is typical of standard alpha topographic maps and has not been observed and reported by any researchers in microstate alpha topographic maps so far. For assessing the association of EEG microstate based frontal hemispheric asymmetry with affect and approach/withdrawal behavior, robust correlation of PANAS and BAS, BIS measures with standard and EEG microstate FA and FTA was estimated. Subsequently, to better understand the neural mechanisms underlying the proposed microstate and standard hemispherical asymmetry measures, they were subjected to the EEG informed fMRI, and their neural underpinnings were estimated. Further, to gain insights into the hemodynamic lateralization associated with the neural underpinnings and its linkage with affect and approach/withdrawal measures, HLI of both asymmetry measures neural underpinnings’ was calculated and subsequently subjected to the robust correlation with PANAS and BAS, BIS measures.

### Robust correlation of frontal hemispherical asymmetry measures with psychological measures

The robust correlation (Pearson, bend, spearman, and skipped) of proposed microstate and standard frontal hemispheric asymmetry measures with PANAS, BIS/BAS psychological scores are tabulated in Table 1.

**Table 1:** Robust correlation (Pearson, bend, spearman and skipped) of standard and proposed microstate based frontal hemispheric asymmetry measures with psychological scores.

EEG alpha frontal asymmetry	Channel pair	Behavioral measure	Pearson Correlation		Bend correlation		Spearman correlation		Skipped correlation			
									Pearson		Spearman	
			r	p	r	p	r	p	r	t	r	t
Standard	F4/F3 (FA)	Positive affect	0.22	0.21	0.2	0.23	0.09	0.54	0.22	1.27	0.09	0.54
		Negative affect	−0.1	0.54	−0.04	0.8	−0.05	0.75	−0.1	−0.6	−0.05	−0.31
		BAS	−0.25	0.37	−0.16	0.56	−0.17	0.56	−0.25	−0.92	−0.17	−0.59
		BIS	−0.03	0.9	0.09	0.75	0.09	0.73	−0.03	−0.12	0.09	0.34
	F8/F7 (FTA)	Positive affect	0.03	0.83	−0.11	0.52	−0.12	0.47	0.03	0.21	−0.12	−0.73
		Negative affect	−0.05	0.75	0.01	0.92	−0.004	0.97	−0.05	−0.31	−0.004	−0.02
		BAS	0.18	0.52	0.17	0.55	0.13	0.65	0.18	0.65	0.13	0.46
		BIS	−0.14	0.62	−0.14	0.61	−0.3	0.28	−0.14	−0.5	−0.3	−1.12
Microstates	F4/F3 (FA)	Positive affect	0.03	0.84	0.08	0.61	0.12	0.46	0.03	0.2	0.12	0.73
		Negative affect	0.35	0.04	0.33	0.05	0.36	0.03	0.35	2.13	0.36	2.2
		BAS	−0.09	0.74	−0.04	0.86	0	1	−0.09	−0.32	0	0
		BIS	−0.3	0.29	−0.41	0.14	−0.28	0.32	−0.3	−1.09	−0.28	−1.01
	F8/F7 (FTA)	Positive affect	0.0003	0.99	−0.01	0.91	−0.01	0.92	0.0003	0.0018	−0.01	−0.09
		Negative affect	0.42	0.01	0.42	0.01	0.38	0.02	0.42	2.64	0.38	2.34
		BAS	−0.17	0.54	−0.18	0.52	−0.18	0.53	−0.17	−0.62	−0.18	−0.64
		BIS	−0.32	0.25	−0.45	−1.7	−0.33	−1.22	−0.32	−1.19	−0.33	−1.22

Standard FA and FTA revealed no statistically significant correlation with PANAS as well as BIS/BAS measures. Similarly, proposed microstate based FA and FTA yielded insignificant low correlation with positive affect score.

**Figure 2:**
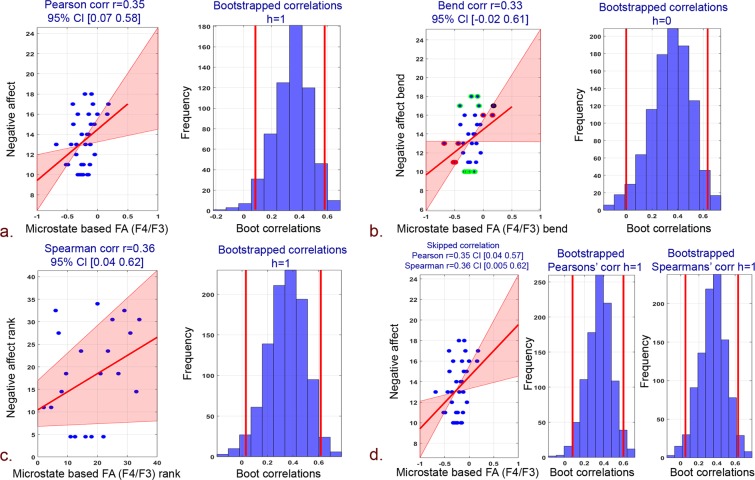
Correlation plots between negative affect scores and microstate based FA (F4/F3) and associated histograms of correlations for bootstrapped data. (**a**) Pearson correlation. (**b**) 20% Bend correlation. (**c**) Spearman correlation. (**d**) Skipped (Pearson and Spearman) correlations.

**Figure 3:**
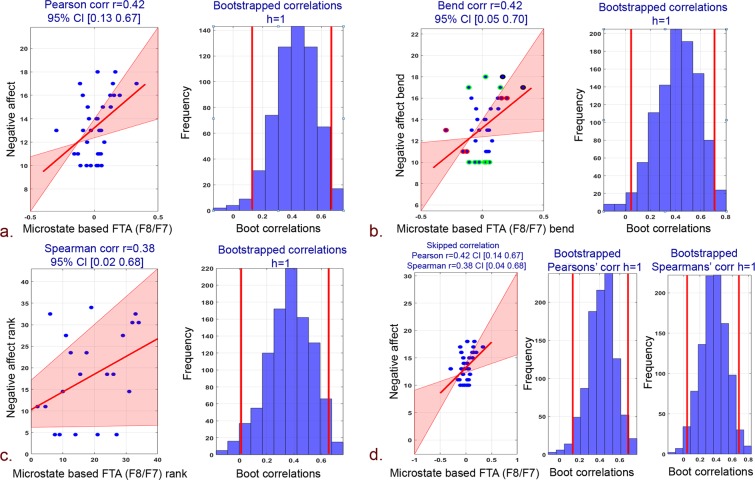
Correlation plots between negative affect scores and microstate based FTA (F8/F7) and associated histograms of correlations for bootstrapped data. (**a**) Pearson correlation. (**b**) 20% Bend correlation. (**c**) Spearman correlation. (**d**) Skipped (Pearson and Spearman) correlations.

Howbeit, negative affect scores revealed a strong and significant correlation with proposed microstate based FA and FTA. Specifically, microstates based FA yielded high pearson, bend and spearman correlations (Fig. 2a: pearson r = 0.35, 95% CI = [0.07; 0.58], pcorr = 0.04; Fig. 2b: bend r = 0.33, 95% CI = [−0.02; 0.61], pcorr = 0.05; Fig. 2c: spearman r = 0.36, 95% CI = [0.04; 0.62], pcorr = 0.03). Similarly, skipped pearson and spearman robust correlations of microstates based FA with negative affect scores has also yielded stronger correlations (Fig. 2d: pearson skipped = 0.35, 95% CI = [0.04; 0.57]; spearman skipped = 0.36, 95% CI = [0.005; 0.62]). In addition, a strong robust pearson, bend and spearman correlation of microstates based FTA with negative affect scores was observed (Fig. 3a: pearson r = 0.42, 95% CI = [0.13; 0.67], pcorr = 0.01; Fig. 3b: Bend r = 0.42, 95% CI = [0.05; 0.70], pcorr = 0.01; Fig. 3c: spearman r = 0.38, 95% CI = [0.02; 0.68], pcorr = 0.02). Skipped (pearson and spearman) correlations among microstates-derived FTA and negative affect scores has also yielded stronger correlations (Fig. 3d: Pearson skipped = 0.42, 95% CI = [0.14; 0.67]; Spearman skipped = 0.38, 95% CI = [0.04; 0.68]).

However, BAS measures yielded a statistically insignificant low correlation with proposed microstate asymmetry. The analysis with BIS measures for both FA and FTA revealed high correlation, but the p-values remained insignificant.

### EEG informed fMRI analysis

The proposed microstate and standard hemispherical asymmetry measures were subjected to the EEG informed fMRI analysis to assess their neural underpinnings, respectively. The observed neural underpinnings were inferred with FDR corrected p-values less than 0.05, and a cluster size of more than 20 voxels were considered for analysis.

### Neural underpinnings of standard hemispheric asymmetry

**Figure 4:**
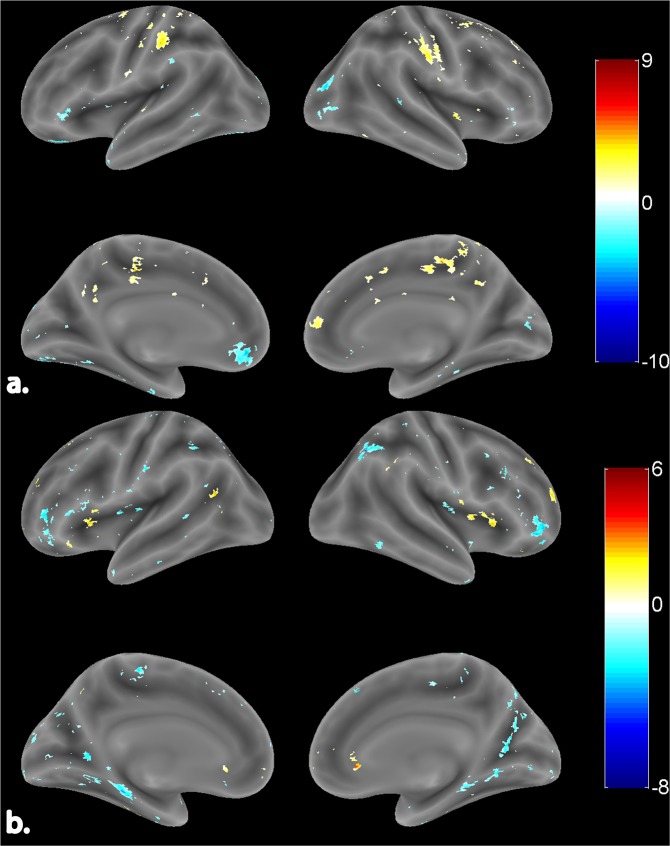
Surface rendered view of neural underpinnings of standard. (**a**) FA (channel pair F4/F3). (**b**) FTA (channel pair F8/F7). The color bar indicates the t-values with blue being the least and red being the highest. The activations are represented at FDR corrected p < 0.05.

Neural underpinnings of standard FA encompassed right as well as left-hemispheric regions (Fig. 4a). Table 2 comprises of these areas, their peak coordinates, and cluster size. Specifically, in the right hemisphere, EEG frontal asymmetry negatively correlated with BOLD activity in occipital cortex with major clusters in lateral occipital cortex and occipital pole. Additionally, BOLD activity in temporal cortex also correlated negatively with standard FA. However, BOLD of parietal cortex regions, particularly postcentral gyrus, correlated positively with standard FA. Withal, in the left hemisphere, standard FA correlated positively with BOLD activity in the postcentral gyrus. However, activity in the occipital fusiform gyrus and temporal lobe regions correlated negatively with this alpha asymmetry measure. Majority of frontal lobe regions correlated negatively. However, superior frontal gyrus correlated positively (high t-value as compared to the right hemisphere) with standard FA.

**Table 2:** Neural underpinnings of standard FA (channel pair F4/F3).

Cluster Center Region	Cluster No.	Voxels	MNI Coordinates			T-Stats
Right Hemispheric Activations						
**Frontal Lobe**						
Superior frontal gyrus	1	43	22	4	48	4.271
**Parietal Lobe**						
Postcentral gyrus	1	246	50	−20	38	6.414
	2	97	16	−28	44	8.087
	3	33	14	−44	60	3.886
	4	23	48	−26	64	−3.349
Superior parietal lobule	1	48	18	−46	64	4.345
**Occipital Lobe**						
Lateral occipital cortex, superior division	1	96	38	−86	14	−4.214
Lateral occipital cortex, inferior division	1	31	36	−72	−30	−2.84
	2	20	46	−80	2	−3.317
Occipital pole	1	96	22	−90	26	−4.04
Intracalcarine cortex	1	27	10	−80	10	−2.747
**Temporal Lobe**						
Temporal pole	1	20	42	14	−32	−2.74
**Limbic Lobe**						
Paracingulate gyrus	1	34	12	50	10	3.121
Insular cortex	1	38	34	−6	−2	9.396
**Left Hemispheric Activations**						
**Frontal Lobe**						
Superior frontal gyrus	1	118	−24	−4	62	7.828
	2	26	−6	56	30	−3.36
Frontal medial cortex	1	117	−12	42	−10	−5.587
Frontal orbital cortex	1	60	−22	26	−18	−6.658
Precentral gyrus	1	60	−16	−26	40	6.636
Inferior frontal gyrus	1	27	−46	30	−2	−3.36
**Parietal Lobe**						
Postcentral gyrus	1	147	−46	−26	38	5.015
	2	36	−36	−28	70	3.992
	3	21	−62	−8	22	2.715
Superior parietal lobule	1	132	−30	−46	64	7.511
**Occipital Lobe**						
Occipital fusiform gyrus	1	159	−22	−84	−10	−3.179
Occipital pole	1	29	−16	−90	30	−3.58
	2	20	−2	−98	0	−2.736
**Temporal Lobe**						
Temporal Occipital Fusiform Cortex	1	159	−28	−66	−22	−2.946
Temporal Fusiform cortex, posterior division	1	39	−36	−14	−26	−3.441
**Limbic Lobe**						
Cingulate gyrus, posterior division	1	34	−8	−54	28	4.73

The activations after correction for multiple comparisons are represented at p < 0.05 (FDR corrected). The coordinates reported are in Montreal Neurological Institute (MNI) space.

Figure 4b shows the neural underpinnings of standard FTA. Both right and left hemispheres revealed positive as well as negative correlations between BOLD activity and standard FTA (Table 3). In the right hemisphere, BOLD activity in occipital lobe regions (cuneal cortex, lingual gyrus, and superior division of lateral occipital cortex) correlated negatively with standard FTA. Major clusters in the frontal lobe, specifically frontal pole, and activity in precuneus cortex also found a negative correlation with this frontal asymmetry index. In the left hemisphere, standard FTA correlated negatively with BOLD activity in Inferior frontal gyrus. Few clusters in parietal, occipital and temporal pole also correlated negatively with standard FTA. The neural underpinnings of standard FA showed left-hemispheric dominance whilst FTA revealed right-hemispheric dominance.

**Table 3:** Neural underpinnings of standard FTA (channel pair F8/F7).

Cluster Center Region	Cluster No.	Voxels	MNI Coordinates			T-Stats
Right Hemispheric Activations						
**Frontal Lobe**						
Frontal pole	1	67	26	54	22	3.822
	2	385	46	38	10	−5.757
	3	385	30	48	−12	−5.113
	4	385	50	44	−10	−2.962
Subcallosal cortex	1	25	6	30	−4	6.522
	2	20	6	14	−4	−3.574
Middle frontal gyrus	1	94	50	14	36	−8.063
Precentral gyrus	1	27	50	6	40	−3.411
**Parietal Lobe**						
Precuneous cortex	1	392	28	−52	10	−4.401
	2	392	22	−66	26	−2.890
Supramarginal gyrus, posterior gyrus	1	36	64	−46	32	3.054
**Occipital Lobe**						
Cuneal cortex	1	392	8	−78	38	−5.546
Lateral occipital cortex, superior division	1	174	34	−62	46	−6.328
Occipital fusiform cortex	1	149	26	−68	−26	−4.764
Lingual gyrus	1	210	14	−58	−4	−2.931
	2	48	2	−76	0	−2.582
Occipital pole	1	20	8	−96	2	−2.553
**Temporal Lobe**						
Inferior temporal gyrus, temporooccipital part		39	56	−54	−14	−3.061
Central operculum cortex		28	36	−12	22	−2.751
**Limbic Lobe**						
Insular cortex		56	30	20	8	3.558
**Left Hemispheric Activations**						
**Frontal Lobe**						
Inferior frontal gyrus	1	305	−50	32	16	−3.912
	2	20	−46	16	26	−2.703
Middle frontal gyrus	1	42	−52	22	30	−3.196
	2	21	−50	14	36	−3.502
Frontal operculum cortex	1	46	−34	18	12	3.808
Precentral gyrus	1	36	−6	−26	52	−2.984
	2	22	−32	−20	72	−3.16
**Parietal Lobe**						
Supramarginal gyrus, posterior division	1	32	−36	−44	36	−2.772
Postcentral gyrus	1	24	−62	−14	36	−2.86
**Occipital Lobe**						
Occipital pole	1	60	−4	−94	22	−2.882
Lateral occipital cortex, superior division	1	26	−8	−86	38	−2.893
Lingual gyrus	1	42	−24	−54	2	−2.896
**Temporal Lobe**						
Temporal pole	1	40	−50	10	−28	−3.633
**Limbic Lobe**						
Parahippocampal gyrus, posterior division	1	175	−10	−38	−22	−5.375
Parahippocampal gyrus, anterior division	1	23	−30	−10	−30	−3.412
Cingulate gyrus, posterior division	1	22	−10	−40	2	−2.716

The activations after correction for multiple comparisons are represented at p < 0.05 (FDR corrected). The coordinates reported are in Montreal Neurological Institute (MNI) space.

### Neural underpinnings of microstate based EEG asymmetry

**Figure 5:**
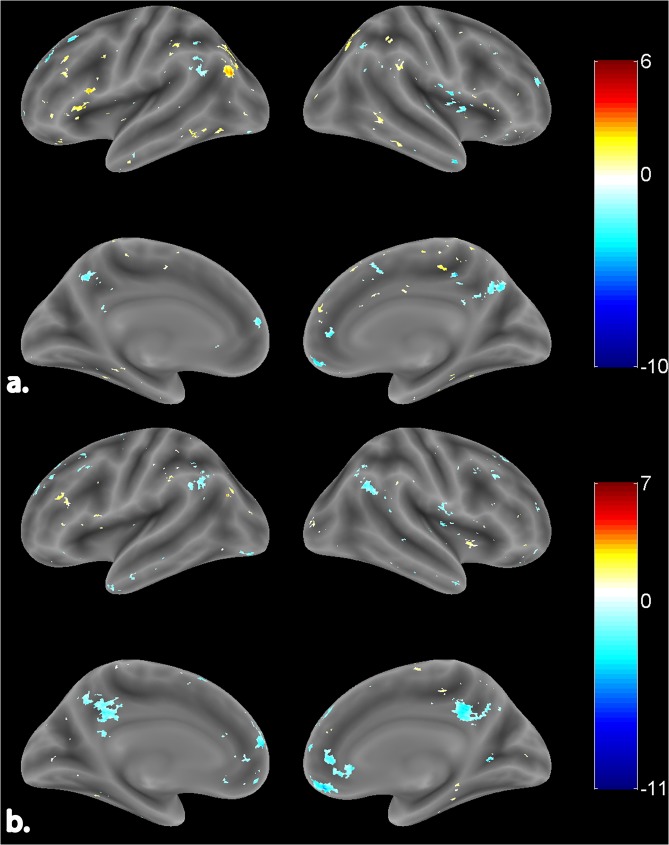
Surface rendered view of neural underpinnings of proposed microstate based. (**a**) FA (channel pair F4/F3). (**b**) FTA (channel pair F8/F7). The color bar indicates the t-values with blue being the least and red being the highest. The activations are represented at FDR corrected p < 0.05.

Right and left-lateralized neural underpinnings of microstate based FA are shown in Fig. 5a. A complete list of activation clusters is provided in Table 4. In the right hemisphere, microstate based FA correlated negatively with BOLD activity in the frontal medial cortex and frontal pole regions of the frontal lobe. Similarly, BOLD activity in the posterior division of cingulate gyrus has also correlated negatively. However, few clusters in the frontal lobe, occipital lobe, and temporal pole revealed a positive correlation with microstate FA. In the left hemisphere, resting-state microstate based FA correlated positively with major clusters in all lobes with frontal lobe having the maximum cluster extent. This is evident as microstates are known to represent the global brain activity.

**Table 4:** Neural underpinnings of proposed microstate based FA (channel pair F4/F3).

Cluster Center Region	Cluster No.	Voxels	MNI Coordinates			T-Stats
Right Hemispheric Activations						
**Frontal Lobe**						
Middle frontal gyrus	1	32	42	34	40	5.93
	2	26	44	4	58	3.035
Superior frontal gyrus	1	24	4	14	60	3.421
Frontal operculum cortex	1	20	40	22	4	3.036
Frontal medial cortex	1	59	4	44	−14	−6.743
Frontal pole	1	20	22	48	18	−3.366
**Parietal Lobe**						
Precuneous cortex	1	23	6	−50	66	4.279
**Occipital Lobe**						
Lateral occipital cortex, superior division	1	64	22	−58	48	4.138
**Temporal Lobe**						
Temporal occipital fusiform cortex	1	60	32	−40	−28	3.691
Middle temporal gyrus, temporoccipital part	1	42	62	−50	−8	4.211
Inferior temporal gyrus,temporoccipital part	1	22	54	−38	−18	3.538
Central operculum cortex	1	22	50	−8	10	−4.769
Middle temporal gyrus, anterior division	1	23	52	0	−36	−5.849
**Limbic Lobe**						
Insular cortex	1	48	40	14	−4	6.748
	2	29	36	2	4	−3.17
Parahippocampal gyrus, posterior division	1	27	36	−28	−10	5.738
Cingulate gyrus, posterior division	1	106	8	−52	28	−3.381
**Left Hemispheric Activations**						
**Frontal Lobe**						
Inferior frontal gyrus	1	82	−54	10	14	5.142
Frontal operculum cortex	1	37	−40	24	6	3.055
Middle frontal gyrus	1	29	−42	30	42	2.89
	2	42	−24	24	36	−7.953
Frontal pole	1	42	−22	40	32	−4.002
Precentral gyrus	1	41	−36	−10	66	−3.66
**Parietal Lobe**						
Supramarginal gyrus, anterior division	1	57	−60	−30	46	3.13
Supramarginal gyrus, anterior division	2	23	−44	−36	44	3.112
Postcentral gyrus	1	23	−14	−38	76	3.168
Supramarginal gyrus, posterior division	1	22	−54	−42	54	3.485
	2	35	−38	−48	36	−3.261
Precuneous cortex	1	144	−4	−58	42	−4.005
**Occipital Lobe**						
Lateral occipital cortex, superior division	1	74	−42	−74	28	4.041
	2	50	−28	−62	30	2.992
	3	41	−30	−78	36	3.427
Lateral occipital cortex, inferior division	1	24	−30	−82	−28	−3.625
Occipital fusiform gyrus	1	21	−34	−86	−20	−3.382
**Temporal Lobe**						
Temporal fusiform cortex, posterior division	1	66	−38	−48	−32	3.132
Inferior temporal gyrus, temporoocipital part	1	23	−58	−54	−14	2.967
**Limbic Lobe**						
Parahippocampal Gyrus, posterior division	1	66	−22	−36	−20	3.035

The activations after correction for multiple comparisons are represented at p < 0.05 (FDR corrected). The coordinates reported are in Montreal Neurological Institute (MNI) space.

Figure 5b shows the neural underpinnings in both right and left hemispheres for resting-state microstate based FTA. Table 5 comprises of these areas, their peak coordinates, and cluster size. In the right hemisphere, activity in the frontal lobe and limbic lobe regions correlated negatively with this EEG alpha asymmetry. BOLD of specific regions of the parietal lobe (Angular gyrus) and temporal lobe (Planum temporale) correlated negatively with microstate based FTA. Pertaining to the left hemisphere, activations in the frontal lobe and limbic lobe (a posterior division of cingulate gyrus) correlated negatively with microstate based FTA. Negative correlation also emanated from BOLD activity in specific regions of the parietal lobe (Angular gyrus, Superior parietal lobule) and lateral occipital cortex of occipital lobe. The neural underpinnings for microstate based FA and FTA showed left-hemispheric dominance.

**Table 5:** Neural underpinnings of proposed microstate based FTA (channel pair F8/F7).

Cluster Center Region	Cluster No.	Voxels	MNI Coordinates			T-Stats
Right Hemispheric Activations						
**Frontal Lobe**						
Frontal medial cortex	1	192	2	42	−12	−11.711
Frontal pole	1	81	8	50	42	−3.819
	2	25	8	58	14	−2.958
Subcallosal cortex	1	68	6	28	−4	−4.043
Superior frontal gyrus	1	23	16	32	56	−3.107
**Parietal Lobe**						
Angular gyrus	1	104	50	−56	28	−3.685
**Occipital Lobe**						
Lingual gyrus	1	20	26	−56	2	−3.676
**Temporal Lobe**						
Planum Temporale	1	124	62	−12	6	−3.416
**Limbic Lobe**						
Cingulate gyrus, posterior division	1	203	4	−44	38	−5.841
Insular cortex	1	124	36	−12	14	−5.241
**Left Hemispheric Activations**						
**Frontal Lobe**						
Middle frontal gyrus	1	25	−42	34	24	3.014
	2	51	−26	20	38	−3.049
	3	25	−42	18	48	−3.022
Superior Frontal Gyrus	1	138	−4	52	36	−3.021
	2	30	−6	40	50	−4.585
	3	21	−2	14	66	−3.298
Frontal pole	1	138	−8	58	14	−6.323
	2	75	−20	52	30	−3.548
	3	21	−20	44	38	−2.748
Precentral gyrus	1	21	−36	−12	68	−2.877
**Parietal Lobe**						
Supramarginal gyrus, anterior division	1	87	−62	−28	40	7.244
	2	41	−44	−36	46	2.927
Angular gyrus	1	181	−46	−56	54	−3.641
	2	181	−58	−54	36	−3.615
Superior Parietal Lobule	1	181	−34	−52	38	−2.478
**Occipital Lobe**						
Lateral occipital cortex, inferior division	1	91	−30	−88	−18	−3.47
**Temporal Lobe**						
Temporal pole	1	47	−44	10	−36	−4.092
**Limbic Lobe**						
Cingulate gyrus, posterior division	1	386	−6	−48	36	−5.096
	2	386	−4	−44	14	−2.506

The activations after correction for multiple comparisons are represented at p < 0.05 (FDR corrected). The coordinates reported are in Montreal Neurological Institute (MNI) space.

### Robust correlation of HLI with PANAS, BIS/BAS measures

The correlation and p-values for all the significant results obtained for this analysis are tabulated in Table 6. The robust correlation between negative affect measure and HLI of neural underpinnings of microstate frontal alpha asymmetry yielded a significantly strong negative correlation in the anterior division of the middle temporal gyrus. Further, superior frontal gyrus emerged as the positive correlate for correlation among positive affect scores and HLI pertaining to neural underpinnings of microstate alpha asymmetry. Moreover, the correlation of BIS measure with HLI pertaining to neural underpinnings of microstate frontal alpha asymmetry yielded a significantly strong positive correlation in inferior frontal gyrus (pars triangularis) and frontal medial cortex. Further, the HLI of occipital fusiform gyrus correlated negatively with BAS measure.

**Table 6:** Robust correlation (Pearson, bend, spearman and skipped) of HLI based on standard and proposed microstate based frontal hemispheric asymmetry measures with psychological scores.

Hemodynamic Lateralization Index (HLI)	Behavioral measure	Cortical regions	Pearson Correlation		Bend correlation		Spearman correlation		Skipped correlation			
									Pearson		Spearman	
			r	p	r	p	r	p	r	t	r	t
Standard neural underpinnings	Negative affect	No region survived	—	—	—	—	—	—	—	—	—	—
	BIS	No region survived	—	—	—	—	—	—	—	—	—	—
	Positive affect	Insular cortex	0.44	0.004	0.44	0.005	0.4	0.01	0.53	3.82	0.41	2.75
	BAS	No region survived	—	—	—	—	—	—	—	—	—	—
Microstates Neural underpinnings	Negative affect	Middle temporal gyrus, anterior division	−0.4	0.01	−0.38	0.01	−0.43	0.006	−0.4	−2.67	−0.43	−2.91
	BIS	Inferior frontal gyrus	0.69	0.005	0.63	0.01	0.69	0.005	0.69	3.36	0.69	3.39
		Frontal medial cortex	0.71	0.004	0.76	0.001	0.75	0.001	0.71	3.53	0.75	4.04
	Positive affect	Superior frontal gyrus	0.36	0.02	0.3	0.05	0.31	0.05	0.36	2.37	0.31	2.01
	BAS	Occipital fusiform gyrus	−0.58	0.02	−0.57	0.03	−0.55	0.03	−0.58	−2.51	−0.55	−2.32

However, the robust correlation between negative affect measure and HLI of neural underpinnings of standard frontal alpha asymmetry yielded low and insignificant correlation with all cortical regions. Whilst correlation of positive affect scores with HLI pertaining to standard alpha asymmetry revealed a significant positive correlation with the insular cortex. Further, the correlation of BAS and BIS measures with HLI revealed a low and insignificant correlation with all cortical regions pertaining to standard alpha asymmetry.

### Robust correlation among frontal hemispherical asymmetry measures

**Figure 6:**
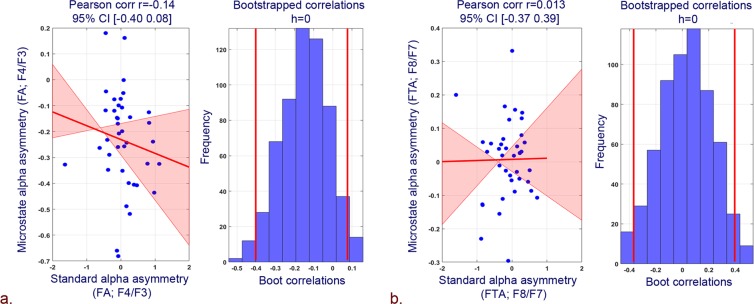
Pearson correlation plots and associated histograms for bootstrapped data for correlation between. (**a**) Standard and microstate based FA (F4/F3). (**b**) Standard and microstate based FTA (F8/F7).

Figure 6 shows the Pearson robust correlation of proposed microstate frontal hemispheric asymmetry with standard frontal hemispheric asymmetry measures. Proposed microstate based FA and FTA yielded insignificant low correlation with standard FA and FTA. Pearson correlation among standard and microstate based FA and FTA revealed correlation coefficients and p-values as Pearson r = −0.14, 0.013; pcorr = 0.37, 0.93 respectively.

## Discussion

Valence^56–59^ and motivation hypothesis^60^ propose that higher values of positive affect/approach behavior and negative affect/withdrawal behavior are associated with the greater relative left and right cortical activation, respectively. These hypotheses are established in task-based EEG alpha asymmetry studies where the implications are based on alpha inhibition (desynchronization w.r.t BOLD) in event-specific regions^45,47^. Thus, following this abstraction, the above-mentioned hypothesis holds when standard frontal hemispheric asymmetry $(\mathrm{ln}({\alpha }^{Right})-\,\mathrm{ln}({\alpha }^{Left}))$ correlates positively with positive affect/ approach behavior and negatively with negative affect/withdrawal behavior. Howbeit, the validity of these hypotheses in resting-state recordings which involves sole perception and not induction of valence/behavior still remains vacillating. The inconsistent results of the relationship between the standard resting frontal asymmetry and affect and approach/withdrawal behavior are tabulated in Table 7. The line of studies by Tomarken *et al*.^29,30^ and Jacob and Snyder^31^ supported the above hypothesis. Similarly, for approach/withdrawal dichotomy, Harmon-Jones and Allen^33^, Shackman *et al*.^61^, and De Pascalis *et al*.^34^ supported the above-mentioned hypotheses. Nonetheless, Sutton and Davidson^35^ and Schneider *et al*.^36^ observed no association of affect, approach/withdrawal dichotomy with frontal asymmetry, respectively. Conversely, the study by Hagemann *et al*.^38^ proposed that subjects with high negative affect exhibited high left cortical activation. Further, Hewig *et al*.^62^ propounded a higher approach measure to be associated with the bilateral frontal cortical activity. Hence, in order to bring more clarity, the present study aims to assess the capability of quasi-stable microstates based frontal hemispheric asymmetry in explaining the affect and approach/withdrawal dichotomy as against standard frontal hemispheric asymmetry.

**Table 7:** List of studies for positive/negative affect and approach/withdrawal dichotomy.

Study	Alpha EEG Asymmetry (R-L)	Mood Measures	Subjects	Main Results
Tomarken *et al*.^136^	FA (F4/F3);	Acquisition of resting EEG followed by the presentation of affective clips to obtain subjective ratings for emotional reactions	32 females, Cohort A: 17 to 41 years Cohort B: 20 to 54 years	Resting FA significantly predicted self-reported global NA
Tomarken *et al*.^29^	FA (F4/F3); ATA (T4/T3)	Resting EEG on two occasions; 3 weeks apart; PANAS	90 females, 17–21 years	FA:↓NA ATA:↑PA
Tomarken *et al*.^30^	Same as in Tokarman *et al*., 1992a	Same as in Tomarken *et al*., 1992a	85 females, 17–21 years	Same as in Tomarken *et al*., 1992a
Jacobs and Snyder,1996^31^	FA (F4/F3); FTA (F8/F7)	Resting EEG on 1-time measurement; PANAS	40 males, 18–53 years	FTA:↓NA
Sutton and Davidson,1997^35^	FA (F4/F3)	Resting EEG on two occasions 6 weeks apart PANAS first session; BIS/BAS scales the second session	46 (23 females) 18–22 years	No correlation between FA and PA, NA, BAS, BIS
Hagemann *et al*.^37^	FA (F4/F3); ATA (T4/T3)	Acquisition of resting EEG followed by the presentation of affective slides to obtain subjective ratings for emotional reactions	37 (22 females: 15 males: Mean age 24.5)	Subjects with greater relative left-sided anterior temporal cortical activation reported more intense NA in response to negative stimuli
Hagemann *et al*.^38^	FA (F4/F3); ATA (T4/T3)	Resting EEG; PANAS	36 (24 females) Mean age 24.7	Subjects with high NA exhibited high left cortical activation at the anterior temporal site
Hall and Petruzzello, 1999^32^	FA (F4/F3)	Resting EEG and measures of physical activity; PANAS	41 (26 females) Mean age 68.7	FA positively predicted PA
Harmon-Jones and Allen, 1997^33^	FA (F4/F3);	Resting EEG from females who scored in the upper or lower third of the distribution of social anxiety scores; BAS,BIS	37 females	FA:↑BAS
Hewig *et al*.^62^	FA (F4/F3); FTA (F8/F7); ATA (T4/T3)	Resting EEG on four occasions; four weeks apart; BAS, BIS	59 (30 females: Mean age 23; 29 males: Mean age 25)	Higher BAS associated with bilateral frontal cortical activity
Shackman *et al*.^61^	FA (F4/F3); FTA (F8/F7)	Resting EEG on two occasions; several weeks apart; BAS, BIS	51 females Mean age 19.5	A significant relationship between BIS and FA. Higher BIS associated with right posterior DLPFC
De Pascalis *et al*.^34^	FA (F4/F3); FTA (F8/F7)	Resting EEG; BAS,BIS	51 females Mean age 24.1	FA:↑BAS, Higher BAS associated with left-sided activation in MFG
Schneider *et al*.^36^	FA (F4/F3);	Two assessments of resting EEG; BAS, BIS	99 (50 females; 49 males aged 10–12 years)	No correlation of BAS, BIS measures with FA

EEG, Electroencephelography;↑Positive correlation;↓Negative correlation; FA, Frontal Asymmetry (F4/F3); FTA, Frontal Temporal Asymmetry (F8/F7); ATA, Anterior Temporal Asymmetry (T4/T3); BAS, Behavioral Activation System; BIS, Behavioral Inhibition System; PA, Positive Affect; NA, Negative Affect; DLPFC, Dorsolateral Prefrontal Cortex; MFG, Middle Frontal Gyrus.

### Prelude to the present research study

This study primarily focuses on exploring the ability of EEG microstates based frontal hemispherical asymmetry measure against standard Davidson’s approach in explaining mechanisms of the resting state affect and approach/ withdrawal behavior. The rationale for examining EEG microstates-derived frontal asymmetry was based on the specific observation that affect and approach/withdrawal measures associated significantly with stable EEG signatures. Microstate analysis estimates the global pattern of coherence across entire EEG channels from temporal EEG data and thus assesses patterns of quasi-stable activities. The interaction within a large scale brain network involves a rapid change in the dynamics of these quasi-stable activity patterns. Further, the neural mechanism associated with any cognitive process generally involves the coordinated activity of many neural assemblies located at different cortexes. Correspondingly, the neural mechanisms of resting-state affect and approach/ withdrawal behavior are also the result of one such coordinated activity of the large scale brain networks.

Thus, in this study, a novel approach is explored, which assesses the frontal hemispherical asymmetry of quasi-stable activity patterns (microstates) from large scale brain interactions of the resting state affect and approach/ withdrawal behavior. These EEG microstates based frontal hemispherical asymmetry measures are further subjected to the EEG informed fMRI analysis to estimate their neural underpinnings. Subsequently, the lateralization index, which measures the hemispherical asymmetry of these large scale brain networks based on their hemodynamic information, is measured and correlated with affect and approach/ withdrawal psychological measures. Lastly, the insights brought by the proposed EEG microstates based approach is compared with the standard EEG asymmetry measures to understand the effectiveness of microstate derived asymmetry measures in explaining resting-state affect and approach/ withdrawal behavior. The insights of the present study are summarized in the following subsections.

### Standard alpha asymmetry and its HLI reveal no correlation with PANAS and BIS/BAS measures

The current study is in line with the observation of Davidson and colleagues^35^ and other earlier studies^4,36,63^, where no correlation was observed for affect and BIS/BAS measures with standard hemispheric asymmetry. However, these previous studies never explored the neurovascular underpinnings and associated hemodynamic asymmetry of these underpinnings. In the present study, the absence of linkage of standard hemispheric asymmetry with affect and BIS/BAS measures is further strengthened by the lack of correlation of HLI of neural underpinnings of standard alpha asymmetry with PANAS, BIS, and BAS measures. This supports the understanding that neural mechanisms that are measured as standard EEG frontal alpha asymmetry may not be the marker to explain the affect and/or approach-withdrawal measures during resting state. It might possibly are influenced by the neural activity associated with other ongoing resting-state neural mechanisms, which limit its sensitivity towards the neural mechanisms associated with affect and approach-withdrawal measures during resting state. Thus, our finding strengthens the understanding that the standard EEG alpha asymmetry model, especially in the male population, is effectual in explaining affect or approach-withdrawal measures only when arousing situations such as those relying on mood induction procedures are present.

### Microstates based asymmetry correlates with and delineates the neural mechanisms of Negative affect

**Figure 7:**
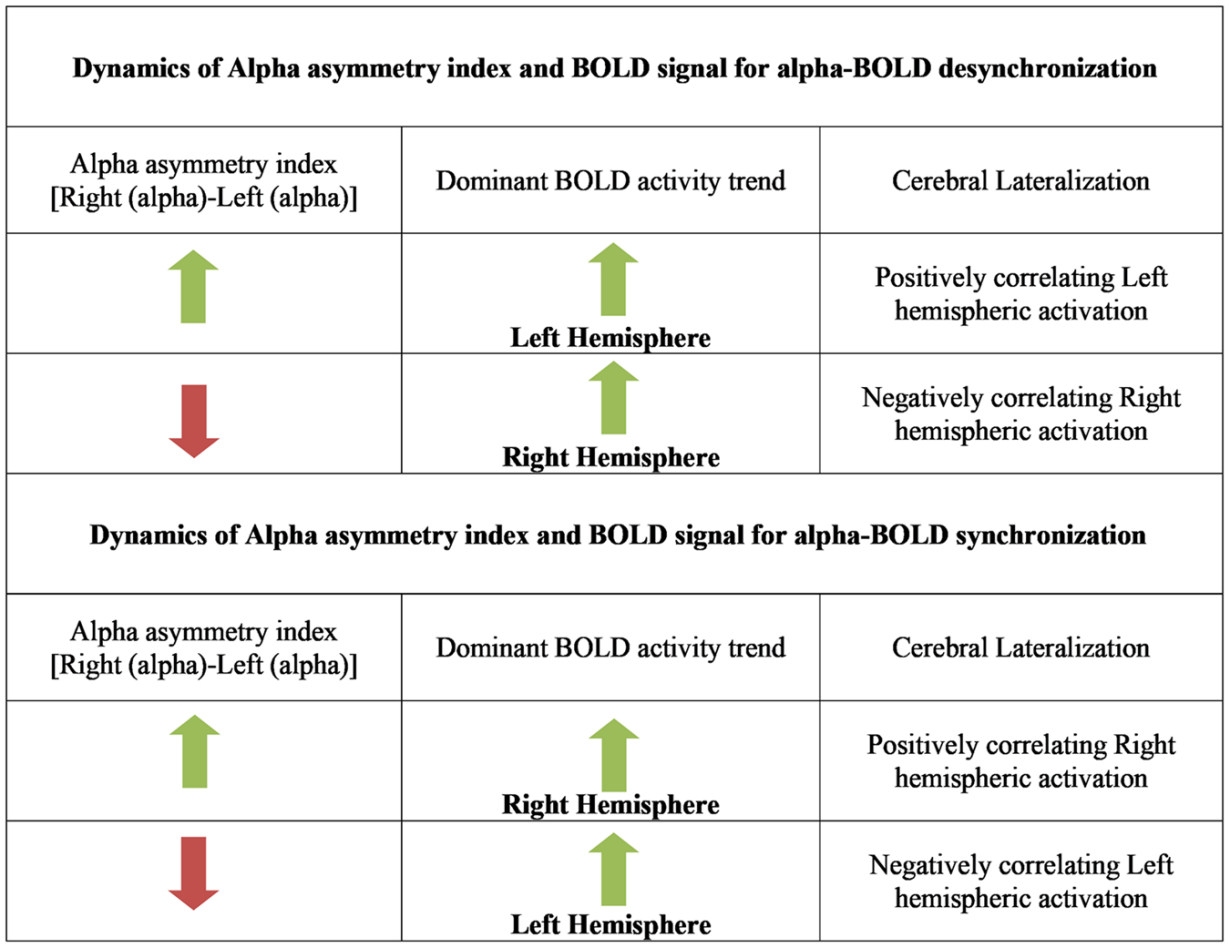
Underlying dynamics associated with alpha asymmetry index and BOLD signal.

In contradistinction to the standard hemispheric asymmetry, the proposed microstates based measure brings better insights into the global coordinated activity of large scale brain networks pertaining to negative affect. In this study, the robust correlational analysis revealed a positive correlation of negative affect with microstates based frontal hemispheric asymmetry. This implies that negative affect increases with an increase in right hemispheric alpha activity or a decrease in left-hemispheric alpha activity. Further, the most common neurovascular hypotheses state that when engaged in the task, the brain region exhibits suppression in alpha power with an increase in BOLD signal^47^. This causes a negative correlation between alpha power and BOLD signal and is termed as alpha-BOLD desynchronization. Figure 7 depicts these underlying dynamics for the association between alpha asymmetry measures and the BOLD signal during alpha-BOLD synchronization/desynchronization. Following this, the positive correlation of negative affect with microstates based frontal hemispheric asymmetry implies left-hemispheric interaction with negative affect. These observations do not support the valence hypothesis explained in the earlier section but goes in line with the observations by Hagemann *et al*.^38^, wherein negative affect has been linked to the left-hemisphere. Our results were also in line with a mood induction study by Gale *et al*.^64^, where negative mood increased with an increase in left frontal activation. Further, recently Farahi *et al*.^65^ showed the associativity of fear positively with the left hemisphere.

Additionally, neural underpinnings of microstate derived asymmetry revealed the involvement of temporal lobe regions. In this study, HLI ($HRF\_Am{p}_{n}^{R}-HRF\_Am{p}_{n}^{L}$), which was estimated by utilizing the amplitude of the HRF of each neural underpinning of microstate based frontal asymmetry linked negatively the HLI of the anterior division of middle temporal gyrus neural underpinning to negative affect. This implies that relatively left-lateralized HRF amplitude of temporal underpinning pertaining to microstate based frontal asymmetry is associated with negative affect. Studies in the past have connected left anterior temporal cortical activation as well as temporal lobe per se to the negative affect;^66–69^ this proves the efficacy of microstate based frontal asymmetry in explaining the neurovascular mechanism of negative affect which remains absent in the previous literature. Batut *et al*.^70^ signaled the involvement of mesial temporal regions in emotional processes; further, Yun *et al*.^71^ showed that the angst for social communication in social anxiety disorder could be resultant of the imbalanced functional connectivity of left middle temporal gyrus. The association of anterior division of middle temporal gyrus with negative affect is plausible as studies^72–74^ have indicated the interaction between middle temporal gyrus and amygdala for better prediction of memory for emotional events. Hence, the middle temporal gyrus may be more tightly functionally coupled with affect specific regions for the memory of negative events. The significant correlation of negative affect with temporal region’s HLI, which is independently measured from resting fMRI data for neural underpinnings of microstate frontal asymmetry and its relative left lateralization, also strengthens the finding of positive correlation of negative affect with microstate based frontal asymmetry measures (FA and FTA).

### Microstate based asymmetry reveals no correlation with BIS, Positive affect, and BAS

Microstate based asymmetry showed a high but insignificant correlation with BIS measure. It also showed no correlation with positive affect and BAS measure. One possible explanation for these findings is the fact that the positive affect scale is a diverse measure with components of joy, interest, and activation. Each one of these components might involve distinct and sometimes even opposite whole-brain activations^75^. Similarly, BAS is also composed of varied components (reward, drive, and fun)^76^. These varied brain activation patterns might not be producing definite patterns at cortical levels to be picked by the alpha power.

### HLI of microstates neural underpinnings reveals significant association with BIS, positive affect, and BAS measures

The hemodynamic lateralization measure of neural underpinnings of the proposed technique revealed a high and positive correlation of BIS in frontal cortical regions. Frontal cortical regions play a very important role in inhibition systems, and it has been one of the cornerstones of neuroscience research^13,77,78^. Further, Fuentes *et al*.^79^ also emphasized the association of individual differences in the behavioral inhibition system with the orbitofrontal cortex. Hence, our results suggest that the HLI, which constitutes the voxel-level hemispheric differences in HRF amplitude of neural underpinnings of microstates based asymmetry better manifests BIS measure. Further, though microstate based alpha asymmetry found no significant correlation with positive affect and BAS measure, the HLI of occipital fusiform gyrus was found to strongly correlate with BAS measure. This is consonant with the hypothesis where the BAS system is proposed to be modulated by occipital cortices^80^. The nature of correlation was strong but negative and thus was inverse of the BIS system. Moreover, positive affect was correlated positively with hemodynamic lateralization measures in superior frontal gyrus. The link of the prefrontal cortex with positive affect is consistent with recent studies. Wager *et al*.^81^ showed the association of the prefrontal cortex with positive affect as compared to negative affect. Similarly, Roy *et al*.^82^ observed more frequent activity was found in the prefrontal cortex during positive as compared with negative feelings. Hence, hemodynamic lateralization measures of microstates neural underpinnings bring better insight into the positive affect and BAS as compared to the standard EEG based hemispherical asymmetry measures.

Interestingly, the neural underpinnings (middle temporal gyrus (anterior division), inferior frontal gyrus, frontal medial cortex) whose HLI revealed significant correlation (r-value) with negative affect and BIS scores have been observed to be undergoing only alpha-BOLD desynchronization process. They were found to be either correlating positively in the left hemisphere or negatively in the right hemisphere. On the other hand, the neural underpinnings whose HLI correlated with positive affect and BAS scores have revealed both alpha-BOLD synchronization and desynchronization. Particularly, superior frontal gyrus, which correlated with positive affect, underwent both alpha-BOLD synchronization and desynchronization. However, the occipital fusiform gyrus correlated negatively in the left hemisphere, which thus undergoes alpha-BOLD synchronization. Thus, the neural mechanisms involved in negative affect/withdrawal in the resting state exhibited only alpha-BOLD desynchronization. On the contrary, the positive affect and an approach relevant region involved both alpha-BOLD synchronization and desynchronization. However, the underlying innate cause of these mechanisms remains elusive and needs to be explored in the future. Thus, our finding implicates that microstates based frontal alpha asymmetry may provide newer insights into the association of alpha asymmetry with mood and personality measures in both healthy and clinical populations. The plausible explanation is that different cognitive states, including affect and approach/withdrawal behavior, generally involve coordinated activity of many neural assemblies located at the different cortex, and the microstate prototypes could represent these cognitive states.

### Absence of correlation among proposed microstate and standard frontal hemispheric asymmetry measures

The proposed microstate based FA and FTA yielded an insignificant low correlation with standard FA and FTA. The proposed microstate based FA and FTA measure the quasi-stable coordinated brain activity and, in the present study, brings better insights into the large scale brain networks of negative affect. Previous works of literature^29,39^ have also emphasized the prominence of stability in the standard EEG patterns in bringing forth the linkage among standard frontal alpha asymmetry and affect and approach/withdrawal measures. Hence, the lack of correlation among proposed microstate and standard frontal hemispheric asymmetry measures might be caused by the unstable nature of standard EEG and its frontal alpha asymmetry indices, which is caused by a substantial interference from many other cognitive factors. As this interference is different at different time points across volunteers, the standard EEG and its frontal alpha asymmetry are likely to correlate less with the quasi-stable patterns assessed by the proposed microstate frontal alpha asymmetry indices.

### Limitation of the study

The present study utilizes 39 volunteers’ data to validate the role of microstate based resting frontal alpha asymmetry in understanding the neural mechanisms of affect and approach/withdrawal behavior. However, affect and approach/withdrawal behavior is known to be elicited by mood induction tasks. Hence, it is necessary to carry out future studies to validate the proposed microstate based frontal alpha asymmetry during such task engagements. Further, the current research involves healthy volunteers from the Indian urban population. Many studies^83–85^ in the past have revealed the association of affect and approach/withdrawal behavior with the cultural, ethnic, and social background of the individuals. Thus, it is required to examine the proposed microstate based frontal alpha asymmetry approach in a larger population dataset, which includes individuals from various cultural, ethnic, and social backgrounds.

Also, the topographies of average-referenced, preprocessed standard EEG are known to represent the posterior alpha than frontal alpha, and these topographies have also been studied in comparison with other referencing schemes^54,86^. However, the microstate analysis employed in the current study uses an average referencing scheme for frontal alpha asymmetry estimation. The present study follows average referencing for microstate analysis as various studies^40,87^ adequately understand the cognitive phenomena through average-referenced microstate estimations. Further, the effect of different EEG referencing schemes on microstate estimations is still not clearly understood. Extensive, systematic work needs to be undertaken to properly understand the role of varying EEG reference montages based microstate analysis in explaining frontal, posterior, and temporal EEG frequency signatures and topographies.

## Conclusion

The above study validates the effectiveness of resting quasi-stable microstate based asymmetry in explaining the neural mechanisms of affect and approach/withdrawal behavior for healthy young male volunteers during 1-time measurement. The novelty of our work emanates from the fact that we estimated the frontal asymmetry of the alpha power from the average GFP amplitude of the quasi-stable microstates topographies, which might reflect the degree of coordination of the neurons underlying alpha-neural underpinnings. Microstate frontal alpha asymmetry correlated positively with negative affect scores, which are defended by the negative correlation of HLI based on microstates’ temporal neural underpinning with negative affect. Further, a significant association of HLI based on microstate neural underpinnings with positive affect, BAS and BIS measures concludes that the neural mechanisms of affect and approach/withdrawal dichotomy are better explained by the synchronized global firing of neurons and on-going activity of entire brain networks as assessed by quasi-stable microstates frontal alpha asymmetry. This study also stands unique in exploring the underlying neurovascular synchronization/desynchronization mechanisms of microstate based frontal asymmetry measures. The analysis revealed that neural underpinnings involved both positively and negatively correlating brain regions, thus satisfying alpha-BOLD desynchronization and synchronization criteria. However, specifically the microstates neural underpinnings whose HLI correlated with negative affect and inhibition involved alpha-BOLD desynchronization, however the positive affect and approach relevant regions involved alpha-BOLD synchronization as well as desynchronization.

## Methods

**Figure 8:**
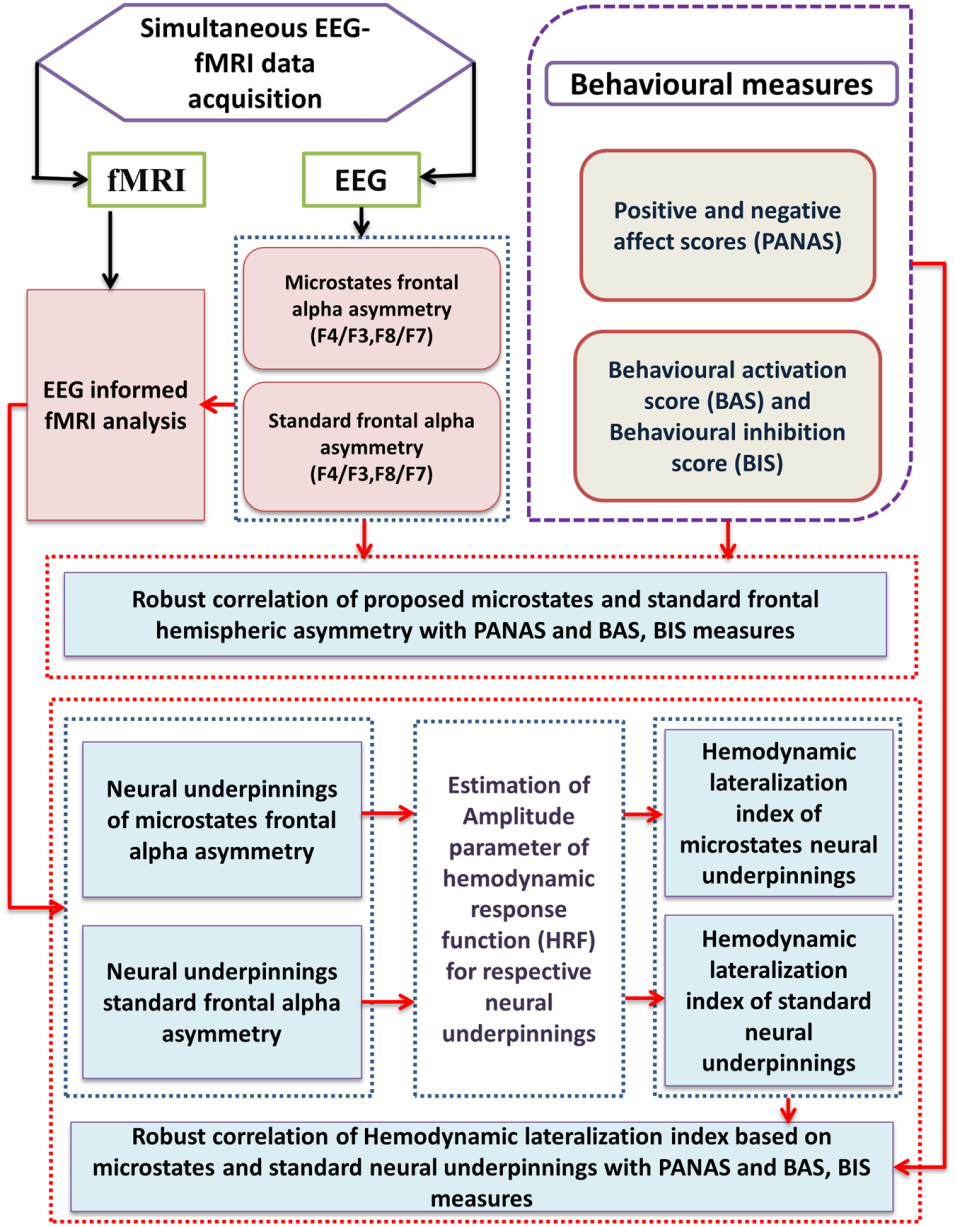
Schema of the methodology adopted in this study.

Figure 8 depicts the schema of the methodology adopted in this study.

### Sample and procedure

Thirty-nine healthy participants (all males; age range 18–24 M = 19.57; SD = 1.28) took part in this study after providing a written and informed consent to the protocol. The experiment has been carried out in accordance with The Code of Ethics of the World Medical Association (Declaration of Helsinki), and all measurements were also approved by the Institute of Nuclear Medicine and Allied Sciences (INMAS) institutional ethical committee (Number: ECR/824/Inst/DL/2016). All subjects were volunteers recruited among university students and were right-handed. Subjects completed a personality questionnaire for positive affect and negative affect and Behavioral inhibition system (BIS)/Behavioral approach system (BAS). The questionnaires were in the English language, and all the volunteers were fluent in the English language. The resting-state fMRI and EEG data analyzed in this paper were collected after the subject completed the psychological questionnaires. The simultaneous EEG-fMRI resting-state recording lasted for 6 minutes with eyes closed condition.

### Behavioral measures

To assess the dispositional affect and approach/withdrawal parameters in resting state, PANAS scores, and BIS/BAS measures were evaluated for each individual. We also estimated the Profile of mood states using POMS scores for prior exclusion criteria. Table 8 presents descriptive characteristics for the study participants with the mean and standard deviation values.

**Table 8:** Demographic and behavioral characteristics of study participants (N = 39).

Variable	Mean (M)	Std. Dev (SD)
Age	19.57	1.28
Positive Affect scores	39.66	5.66
Negative Affect scores	14.64	4.29
BAS scores	23.42	3.5
BIS scores	15.28	2.7

### Positive and negative affect

Positive and negative affect scores were evaluated for each volunteer. Positive and Negative Affect Schedule (PANAS) consists of mood scales designed to assess affect at the present moment^88^. These scales are highly uncorrelated, stable over time, and consistent, and both scales demonstrate good convergent and discriminant validity^89,90^. Positive and negative affect scores showed good internal consistency in our study (Cronbach’s alphas = 0.89; 0.91).

### Behavioral approach system (BAS)/behavioral inhibition system (BIS)

BIS and BAS scores were calculated for each subject^91^ and evaluation included 24 items (20 score-items and four fillers, each measured on four-point Likert scale), and two total scores for BIS (range = 7–28; 7 items) and BAS (range = 13–52; 13 items). In our study, BIS and BAS scales showed good internal consistency (Cronbach’s alphas = 0.93; 0.92).

### POMS (Profile of mood states)

Volunteers were also asked to fill in forms for the POMS^92^. It measures six different dimensions of mood swings, namely Tension or Anxiety, Anger or Hostility, Vigor or Activity, Fatigue or Inertia, Depression or Dejection, Confusion, or Bewilderment. These scores formed the basis for exclusion criteria. All selected volunteer returned self-report scores for all the modes within a relatively normal range.

### Simultaneous EEG-fMRI data acquisition and preprocessing

MRI data was acquired in a Siemens 3 T scanner. After acquiring a high-resolution T1-weighted anatomic rapid gradient-echo image (T1 MPRAGE sequence TR 1900ms, TE 2.49 ms, FA 9°, 160 slices with slice thickness 0.9 mm and distance factor of 50%, FoV 240 mm with voxel size 0.9 × 0.9 × 0.9 mm), we acquired 205 T2*-weighted EPI images for resting-state eyes-closed condition (T2* EPI sequence: TR 2000ms, TE 30 ms, FA 90°, 30 slices with thickness 5 mm and distance factor 0%, FoV 240 mm with voxel size 3.8 × 3.8 × 5.0 mm). Continuous EEG data were acquired simultaneously during resting state T2* acquisition using a 32-channel MR-compatible brain vision cap. The electrodes were placed according to the international 10–20 system with a separate electrode called the Reference electrode, placed between Fz and Cz electrodes, that provided the reference for recording the data. Electrocardiogram (ECG) was also recorded. The impedance level for each electrode was kept less than 5 KΩ. The recorded EEG signal was digitized and transmitted with a sampling frequency of 5000 Hz. The acquisition of EEG signals was accomplished using Brain vision analyzer software.

The fMRI data preprocessing for 205 resting-state volumes was done using the default preprocessing pipeline for volume-based analysis in CONN software. The pre-processing procedure included the realignment and unwarping of T2*-weighted image with the mean functional image for motion correction followed by the translation of center to (0, 0, 0) coordinates and slice time correction of functional data. Functional outlier detection (ART- based identification of outlier scans for scrubbing) was performed, followed by segmentation and direct normalization to MNI space. Next, functional smoothening with a Gaussian Kernel with FWHM of 6 mm was carried out. Further, translation of structural center to (0, 0, 0) and simultaneous structural segmentation and normalization were performed.

EEG data were corrected for gradient artifact using the Brain vision analyzer’s^93,94^ average artifact subtraction algorithm (AAS)^95,96^. A template from MR scanner artifacts was created by averaging the MR scanner artifacts over fixed intervals which were accurately specified by utilizing the fMRI volume markers (labeled as ‘TR’). Subsequently, this average was subtracted from the EEG data. Further, the gradient artifact removed data accommodated six seconds of data prior to the start of the first fMRI block acquisition (identified by the first TR marker). These six seconds is the time the fMRI pulse sequence prepares itself before acquiring the first fMRI block. This prior time interval accommodated gradient-contaminated ECG; hence we truncated these 6 seconds prior data and subjected only the data pertaining to the fMRI volumes to the subsequent cardio ballistic (CB) artifact removal. The CB artifact removal was performed in the FMRIB plugin. The method detects the QRS peaks in the ECG data using combined adaptive thresholding^97^ and Teager energy operator^98^, followed by a correction algorithm. Further, the removal of the CB artifact is performed based on the Optimal Basis Set (OBS) method^99^.

In addition, we also employed the HAPPE toolbox^100^ for further ensuring the quality of conventional EEG artifact removal from the scanner and CB artifact corrected datasets. The following steps utilizing the HAPPE toolbox were adopted. First, the scanner and CB artifact removed data were subjected to the filtering process with 0.1 Hz high pass and 70 Hz low pass filtering, and all the EEG channels were selected for further analysis. This was followed by removal of the electrical (line) noise using the Cleanline plugin^101^ of EEGLAB. The functionality of HAPPE was utilized next to identify and remove the contaminated channels. HAPPE identifies the contaminated channels by evaluating the normed joint probability of average log power across all the channels and rejecting the channels whose joint probability is more than three standard deviations. Wavelet enhanced ICA (W-ICA) approach was implemented subsequently to correct for EEG artifact while retaining the entire length of the data file. The W-ICA approach removes ocular and muscle-related artifacts and also improves the decomposition of later performed ICA, which eventually rejects artifact components. Next, independent components (ICs) with the extended infomax independent component analysis (ICA) were computed, and the MARA plugin^102,103^ of EEGLAB was employed for automatic component rejection. MARA evaluates each component on six features and eventually assigns a probability of artifact contamination to that component. Further, HAPPE’s pipeline automatically rejected any components with artifact probabilities higher than 0.5. Subsequently, segmentation of data based on the markers, rejection of segments, and interpolation of removed channels were carried out. Finally, the processing report about the quality of data was generated. The EEG preprocessing procedures in this study have been explained in detail in Supplementary methods and discussion section. Further the processing report about the quality of data for all volunteers has been tabulated in Supplementary Table 1.

To ensure the quality of preprocessing, we also subjected both raw and final artifact removed EEG data (CSD referenced) to the estimation of the power spectrum between 0.2 Hz to 50 Hz frequency range. The median power spectrum plots of both raw and final artifact removed EEG data (CSD referenced) for channels F3, F4, F7, F8, Pz, Oz, and POz are shown in Supplementary Figs. 1 and 2 respectively. The median spectral power of artifact removed EEG data clearly reveals parietal and occipital alpha and beta bands. Data was down-sampled to 250 Hz for further analysis.

### Assessment of frontal hemispherical asymmetry measures

The main objective of the study was to understand the neural mechanisms associated with the affect, approach/withdrawal behavior, as explained by the hemispherical asymmetry measures. For this purpose, the present study proposes an EEG microstate based frontal hemispheric assessment approach and aims to compare its advantage over the standard EEG frontal asymmetry approach. The following subsections explain the methods for estimating the proposed EEG microstate based frontal hemispheric asymmetry as well as the standard frontal EEG asymmetry.

### EEG microstates based estimation of hemispheric asymmetry

Many recent studies^40,104,105^ have clearly indicated that individual brain functions involve massive parallel processing in distributed brain networks. These distributed brain networks are observed as the scalp field potential in EEG, and the state of global neural activity is measured as a topographical map at that moment of time. The changes in this topography reflect changes in the global coordination of neural activity over time. EEG microstates were proposed to represent changes in behavior, thoughts, and emotions and can be classified into few topographies, which have explained 90% of the variance of continuous EEG. Microstate analysis considers millisecond time range signal from all electrodes to create a global picture of a functional state during that interval.

**Figure 9:**
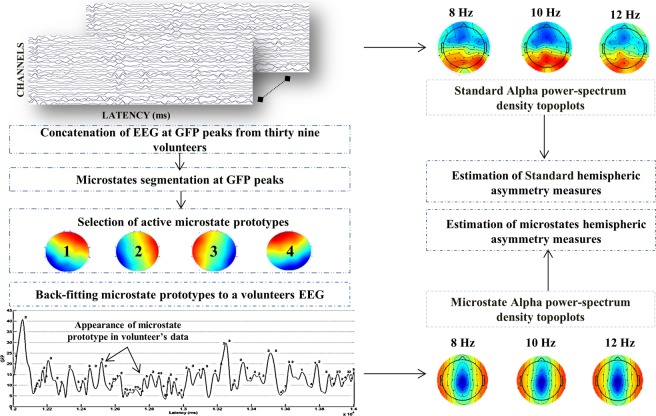
Schema of the methodology adopted for proposed microstate estimation and assessment of standard and microstates based frontal alpha hemispheric asymmetry measures.

The schema of the methodology adopted for microstate estimation is explained in Fig. 9. The aim of a microstate analysis is first to segment EEG maps into microstate prototypes and second to re-express the spatial-temporal characteristics of the time series of EEG through these microstate prototypes.

In this study, let X be the time series EEG information that was acquired from the volunteers. At first, the EEG data X has been pre-processed for removing the artifacts and was referenced to the average referencing. Then, it was subjected to the estimation of Global field power (GFP). GFP is the measure of global brain response to an event and is represented as:

1 $${\rm{GFP}}=\sqrt{(\mathop{\sum }\limits_{i=1}^{C}{({X}_{i}(t)-{X}_{mean}(t))}^{2})/C}$$

where ${X}_{i}$ is the measured potential at the i^th^ electrode at a given time-point t, ${X}_{mean}\,$is the mean value of all ${X}_{i}$’s and C represents the total number of channels. GFP, therefore, represents the standard deviation of the electrode values and indicates, on average, how strong potential is being recorded across the electrode montage^106^. For each volunteer, a selection of data points for the further processing has been carried out by filtering estimated GFPs based on minimum peak distance of 20 milliseconds, and the threshold amplitude of one standard deviation of estimated GFP. Then, the filtered EEG data points of every individual are concatenated to form the GFP datasets for further clustering process as follows:

2 $$\chi =\{{{x}_{GFP}}^{1},{{x}_{GFP}}^{2},\ldots \ldots \ldots .{{x}_{GFP}}^{S}\}$$

where $\chi$ is of the concatenated GFP dataset and ${{x}_{GFP}}^{i}$ are selected data points based on the GFP criteria of the i^th^ volunteer, and S is the total number of volunteers. In this study, thirty-nine volunteers dataset has been subjected to analysis.

Further, concatenated GFP dataset $\chi$ was subjected to the clustering process through the modified K-means clustering algorithm^107^. Modified K-means clustering algorithm requires the initialization of both number (K) of microstate prototype vectors and their components values^108^. Thus, the clustering algorithm was randomly initialized with a set of microstate prototype vectors as the center of initial clusters as follows:

3 $$Z=\{{z}_{i}|i=1\,to\,K\}$$

where K is the total number of microstate prototype vectors (cluster center). In this study, the K is initialized with 8. The clustering algorithm was allowed to iterate and minimize the orthogonal euclidean distance between the data points in $\chi$ as given below.

4 $${\tau }_{n}=arg\mathop{\min }\limits_{k}\{{D}_{kn}^{2}\}$$

5 $${D}_{kn}^{2}={\chi }_{n}^{T}.{\chi }_{n}-{({\chi }_{n}^{T}.{z}_{k})}^{2}$$

where ${\tau }_{n}$ represents the microstate label for n^th^ sample, ${\chi }_{n}\,$represents the n^th^ time sample of the concatenated dataset, ${z}_{k}$ represents the prototypical map for the k^th^ microstate cluster and ${D}_{kn}$ represents the distance between ${\chi }_{n}$ and microstate *k* for the n^th^ time sample. Thus, this clustering algorithm allocates each EEG sample to the cluster whose prototype it is most similar to and then re-estimates microstate prototypes by averaging newly assigned samples^107^. The maximum number of iterations was set to 1000, and the threshold for convergence was set at 1e^−6^ for analysis in this study.

Subsequently, a review of goodness of fit and selection of active microstates is carried out based on global explained variance (GEV) and cross-validation (CV) criterion. It basically evaluates how well microstate segmentation explains the EEG data, which has been used to estimate the prototypes. Therefore, GEV measures how similar the EEG sample and the microstate prototype are; and is calculated as follows.

6 $$GE{V}_{n}=\frac{{(Corr({\chi }_{n},{z}_{{\tau }_{n}}).{x}_{GF{P}_{n}})}^{2}}{{\sum }_{n{\rm{{\prime} }}}^{N}{x}_{GF{P}_{n{\rm{{\prime} }}}}^{2}}$$

where ${\chi }_{n}\,$represents the n^th^ time sample of the concatenated dataset, ${z}_{{\tau }_{n}}$ (${\tau }_{n}=k)\,$is the prototypical map for the k^th^ microstate cluster and ${x}_{GF{P}_{n}}$ represents the n^th^ time sample of the GFP data, and N represents the total number of time samples in concatenated dataset $\chi$. GEV is thus the correlation between the EEG dataset and associated microstate prototype weighted by the EEG dataset’s fraction of the total squared GFP^107^. Thereafter to calculate the GEV for a given cluster, the GEV of its members is summed. Subsequently, CV which is a measure related to the residual noise $\in$ is estimated as,

7 $$CV={\sigma }^{2}.(\frac{C-1}{C-K-1})$$

8 $${\sigma }^{2}=\frac{{\sum }_{n}^{N}{\chi }_{n}^{T}.{\chi }_{n}-{({\chi }_{n}^{T}.{z}_{k})}^{2}}{N(C-1)}$$

where ${\sigma }^{2}$ is the variance of the residual noise, C is the number of EEG channels, N represents the total number of time samples in concatenated dataset $\chi$, and K is the number of clusters. The aim is to obtain a low value of CV. The active microstate prototypes obtained in this study are consistent with the normative EEG microstate classes identified by many studies^40,41,87,109–111^.

Following the selection of an active number of microstate prototypes, the EEG of each volunteer is re-expressed as a sequence of microstate classes by back-fitting these active microstate prototypes on each volunteer’s EEG data. Back fitting implies assigning microstate labels to the EEG dataset based on the dataset’s topographic similarity with the microstate prototype. The estimated re-expressed back fitted dataset is represented as follows

9 $${X}_{re-expressed}=\{{\mu }_{n}|where\,{\mu }_{n}\in \,{Z}_{k{\prime} }\}$$

$${\rm{where}}\,{\mu }_{n}=arg\,{\rm{\min }}(GM{D}_{n})\,$$

The global map dissimilarity (GMD) index measures the topographical similarity between each microstate prototype vector with the EEG sample vector. The GMD is calculated as,

10 $$GM{D}_{n}=\frac{\Vert \frac{{X}_{n}}{{X}_{GF{P}_{n}}}-\frac{{z}_{k{\prime} }}{{z}_{GF{P}_{k{\prime} }}}\Vert }{\sqrt{C}}$$

where ${X}_{n}\,$represents the n^th^ time sample of the preprocessed dataset, ${z}_{k{\prime} }$ represents the prototypical map for the k^th^ microstate cluster. In an ideal condition, if the microstate prototype vector and the EEG sample vector of interest are having the same topographic distribution, then the GMD index will be zero. In case, if both the vectors are topographically opposite, then GMD index would be positively higher. Hence, in this study, instead of the thresholding the GMD index, the microstate prototype vector, which yields a very less GMD index, is chosen as the label for that particular EEG sample vector. Finally, microstates statistics using labels obtained from back-fitted prototypes were calculated.

Subsequently, the amplitude of the microstate prototype vector associated with each label in microstate re-expressed EEG data of every individual is subjected to the alpha band power (8–12 Hz) estimation. The estimated alpha power map of the microstate re-expressed EEG data was used to estimate EEG microstate based frontal hemispheric asymmetry as follows:

11 $$Asymmetr{y}_{MS}=\,\mathrm{ln}\,(\alpha ({{X}_{re-expressed}}^{Right})-\,\mathrm{ln}\,(\alpha ({{X}_{re-expressed}}^{Left})$$

$\alpha ({{X}_{re-expressed}}^{Right})$ and $\alpha ({{X}_{re-expressed}}^{Left})$ are the alpha powers measured at the right and left hemispheric channel of microstate re-expressed EEG data, respectively.

### Standard EEG estimation of hemispheric asymmetry

In order to estimate standard frontal asymmetry, the preprocessed EEG data is first re-referenced to CSD reference using the CSD toolbox^112,113^. Recent work suggests that the CSD transformation reduces the influence of non-frontal sources to frontal asymmetry and may provide a better index of individual differences in frontal asymmetry^114^. Subsequently, the power spectral density (PSD) of alpha frequency (8–12 Hz) was extracted. The estimated alpha power map EEG data was used to calculate standard EEG frontal hemispheric asymmetry as follows:

12 $$Asymmetr{y}_{Standard}=\,\mathrm{ln}\,(\alpha {(X)}^{Right})-\,\mathrm{ln}\,(\alpha {(X)}^{Left})$$

$\alpha {(X)}^{Right}$ and $\alpha {(X)}^{Left}$ are the standard alpha powers measured at the right and left hemispheric channels of individual EEG data, respectively.

Table 9 presents the median and median absolute deviation values for EEG asymmetries for mid-frontal and lateral-frontal sites.

**Table 9:** Median and median absolute deviation of the standard and proposed microstates based frontal hemispheric asymmetry measures.

Variable	Channel pair F4/F3 (FA)		Channel pair F8/F7 (FTA)	
	Median	Median Absolute Deviation	Median	Median Absolute Deviation
Standard hemispheric asymmetry^a^	0.0347	0.3509	−0.052	0.3655
Microstates based hemispheric asymmetry^a^	−0.2324	0.1427	0.0256	0.0896

^a^The difference between log-transformed alpha values from one right-hemispheric electrode to the corresponding electrodes on the left.

### Robust correlation of frontal hemispherical asymmetry measures with psychological measures

Further, estimated EEG microstate and standard frontal hemispherical asymmetries are correlated with PANAS and BAS, BIS measures. These robust correlations were carried out for hemispherical measures that are estimated for both channel pairs F4/F3 i.e. Frontal Asymmetry (FA) and F8/F7 i.e. Frontal Temporal Asymmetry (FTA) independently. The rationale for choosing these channels was based on the linkage of hemispheric asymmetry to mid-frontal (F3, F4) and lateral frontal (F7, F8) sites^39,60,115^. Robust correlations were implemented in Robust correlation Matlab toolbox^116^. This method detects and protects against any bivariate or univariate outliers. Pearson, Bend, and Spearman correlation coefficients, as well as bootstrapped confidence intervals, were computed to evaluate each correlation. Both p-values and confidence intervals were Bonferroni corrected for multiple comparisons.

### Assessment of neural mechanisms associated with functional hemispheric asymmetry measures

One of the focuses of the present study is to understand the neural mechanisms associated with proposed and standard functional hemispheric asymmetry measures in explaining the affect and approach/ withdrawal behavior during resting state. For this purpose, both proposed and standard hemispheric asymmetry measures were subjected to the EEG informed fMRI, and their neural underpinnings were estimated. Subsequently, the lateralization index based on differences in the amplitude of hemodynamic response of neural underpinnings of both hemispheric asymmetry measures was assessed. Finally, the estimated lateralization index was correlated with PANAS and BAS, BIS psychological measures to understand the ability of both hemispheric asymmetry measures in explaining affect and approach/ withdrawal behavior during resting state. The following sub-sections explain these operations in detail.

### EEG informed fMRI analysis

Estimation of neural underpinnings of proposed microstate based EEG asymmetry and standard asymmetry was carried out as follows. At first, the estimated alpha powers for frontal channels F3, F4 F7, and F8 were down sampled to match the acquisition blocks of fMRI (TR: 2 seconds). This was carried out by taking the median of the alpha powers for these specific channels corresponding to each fMRI scan time, which is 2 seconds. The onset time of EEG and fMRI acquisition were also matched. This yielded one EEG alpha power corresponding to each fMRI scan, respectively. Thereafter, microstate based and standard FA and FTA were estimated. The first-level analysis in the present study was performed in SPM12. Different design matrices were obtained each for microstate based and standard asymmetry respectively for each subject wherein microstate based and standard FA and FTA parametrically modulated the fMRI regressors in EEG informed fMRI analysis^117–121^.

The first-level analysis in our study was performed in SPM12, and the time series of fMRI regressors and parametric modulators were convolved with canonical HRF and with its time and dispersion derivatives. Further, at first-level, an F-contrast was defined for parametric modulators subsuming both non-derivative (canonical HRF) and derivative terms (time and dispersion derivatives) for microstate based FA, standard FA, microstate based FTA and standard FTA models.

Subsequently, for the second level of analysis, the first-level contrast images, along with the dispersion and temporal derivatives, were subjected to extraction of amplitude measures from the basis sets^122–126^. The robust regression toolbox^127^ was used to conduct group-level random-effects analysis. The robust regression toolbox uses iteratively re-weighted least squares (IRLS), which detects influential extreme outliers. Thus, the IRLS analysis reduces the likelihood of false-positive and negative findings with no reduction in power and minimizes the effect of extreme outliers^128^. The IRLS has proved beneficial with small samples (n = 10), and the benefits tend to increase with larger sample sizes (n = 40). Further, IRLS controls false-positive rates at an appropriate level when no true effects are present. The contrast image for amplitude summary measure was then subjected for the whole brain analysis corrected with voxel-wise False Discovery Rate (FDR) thresholded at q < 0.05. This yielded the underpinning of both microstate based FA and FTA and standard FA and FTA.

### Estimation of Hemodynamic lateralization index and its robust correlation with psychological measures

The lateralization index measures the hemispherical dominance within the large scale brain network that integrates the neural underpinnings associated with resting affect and approach/withdrawal behavior. The neural activity associated with the neural underpinnings of each hemisphere causes differential electrical potential on the cortical surface of the respective hemisphere. This is measured as the EEG asymmetry index, as explained in the earlier sections. In the mean-time, these differential neural activities of each hemisphere generate a feed-forward signal, which results in differential hemodynamic response at the location of neural activity. Measurement of these hemodynamic hemispherical differences facilitates a better understanding of hemispherical dominance within the large scale brain interactions. Diverse methods have been proposed to calculate the hemodynamic lateralization index on the basis of fMRI BOLD information. As most of these studies involved task engagement, the hemispherical difference of cluster size and BOLD signal strength^129–133^ were normally used to estimate the HLI.

The main motivation behind this estimation is to understand whether hemodynamic asymmetry reveals more insight into understanding the neurovascular mechanisms of the affect and approach /withdrawal behavior. For this purpose, initially, we estimated the hemodynamic response function metric that is hemodynamic response function amplitude (HRF_Amp) at every voxel by independently subjecting the preprocessed resting fMRI data to blind deconvolution method as proposed by Wu *et al*.^134,135^. The estimation of HRF was carried out independently by assuming acquired fMRI BOLD signal $y(t)$ as the convolution of neural states $n(t)$ with $\,HRF(t).\,$This is represented as,

13 $$y(t)=conv(n(t),HRF(t))+\in (t)$$

where $\in (t)$ is the noise in the measurement. Further, $n(t)$ is substituted by a hypothetical neural activation model:

14 $$\hat{n}(t)=\mathop{\sum }\limits_{\tau =0}^{\infty }\delta (t-\tau )$$

where $\delta (t-\tau )$ is the delta function. This allows fitting $HRF(t)\,$according to $\hat{n}(t)$ using a canonical HRF and two derivatives (temporal and dispersion derivatives). This model is subjected to blind deconvolution approach for retrieving the hemodynamic response function $(HRF(t))\,$of every voxel. Once $HRF(t)$ is obtained, an approximation of $\tilde{n}(t)$ can be calculated using the inverse Fourier transform (deconvolution). Then, $HRF(t)\,$was utilized to estimate the HLI for the neural underpinnings of both microstate based FA and FTA and standard FA and FTA, all considered together. Hence, the cluster results of EEG informed fMRI were used only for the selection of regions for estimating HLI as follows,

15 $$HLI(n)=HRF\_Am{p}_{n}^{R}-HRF\_Am{p}_{n}^{L}$$

where $HRF\_Am{p}_{n}^{R}$ and $HRF\_Am{p}_{n}^{L}$ are the median amplitude of hemodynamic response function of the n^th^ neural underpinnings in the right and left hemispheres, respectively. The median of estimated HLI of neural underpinnings of proposed microstate based EEG asymmetry and the standard EEG asymmetry measures were finally subjected to the robust correlations with PANAS and BIS/BAS measures.

## Supplementary information

Supplementary information.

## Data Availability

The data for this study is available from the corresponding author on a reasonable request.
