# Supplementary material for: Microstates-based resting frontal alpha asymmetry approach for understanding affect and approach/withdrawal behavior
Source: Sci Rep. 2020 Mar 6;10:4228. doi: 10.1038/s41598-020-61119-7 (PMC7060213; doi:10.1038/s41598-020-61119-7)
Supplement: Supplementary file 1 — Supplementary information. [file 41598_2020_61119_MOESM1_ESM.docx]

**Microstates-based resting frontal alpha asymmetry approach for understanding affect and approach/withdrawal behavior**

Ardaman Kaur^a,b^, Vijayakumar Chinnadurai^*a^, Rishu Chaujar^b^

a. NMR Research Centre, Institute of Nuclear Medicine and Allied Sciences, Lucknow Road, Timarpur, Delhi-110054, India. b. Department of Applied Physics, Delhi Technological University, Shahbad Daulatpur, Main Bawana Road, Delhi-110042, India.

***Corresponding author**

Dr. Vijayakumar Chinnadurai,

Scientist 'F',

NMR Research Centre,

Institute of Nuclear Medicine and Allied Sciences,

Lucknow Road, Timarpur,

Delhi-110054, India

E-mail: [vijayakumar@inmas.drdo.in](mailto:vijayakumar@inmas.drdo.in), [vijayafmc@gmail.com](mailto:vijayafmc@gmail.com)

Contact no.: +91 9643310095

**SUPPLEMENTARY METHODS AND DISCUSSION**

In the present study, the EEG preprocessing procedures have been meticulously followed to ensure the quality of the preprocessed dataset. This supplementary methods and discussion briefly explain the crucial steps involved in those EEG preprocessing procedures.

1. **Utilizing Average Artifact Subtraction (AAS) based MR gradient artifact removal algorithm in the brain vision analyzer**

Supplementary Fig. 1 shows the median power spectrum plots (0.2 to 50 Hz) of raw EEG data with MR gradient artifact peaks observed at the 15, 30, 45 Hz, and so on (i.e., multiples of no. of slices/TR) for channels F3, F4, F7, F8, Pz, Oz, and POz. To remove gradient artifact and mitigate any residuals at the 15, 30, 45 Hz range, we employed the AAS method^1,2^ of the Brain vision analyzer^3,4^. The AAS algorithm primarily operates by building a template of MR scanner artifacts in EEG data by averaging a fixed number of intervals. Moreover, we used the fMRI volume markers (labeled as 'TR') for forming the aforementioned MR gradient template. Since these markers are regularly spaced and accurately identify the starting point of each MR acquisition, the utilization of volume markers as the reference aids in forming a better MR gradient template. This step yielded gradient artifact removed data with no residuals at 15, 30, 45 Hz.

1. **Truncation of Prior EEG data before Cardio Ballistic (CB) correction:**

Our raw, as well as gradient artifact removed data accommodated 6 seconds of data prior to the start of the first fMRI block acquisition (identified by the first TR marker). These 6 seconds is the time the fMRI pulse sequence prepares itself before acquiring the first fMRI block. During these 6 seconds, there would be a gradient effect on both EEG and ECG data. This can cause a reduction in the quality of CB artifact cleaning, as the contaminated ECG of the prior 6 second period would also be used for forming the template. Hence, we chose to remove these 6 seconds prior data and subjected only the data of the fMRI volumes to the CB artifact removal. The CB artifact removal was subsequently performed using the FMRIB plugin. The method detects the QRS peaks in the ECG data using combined adaptive thresholding^5^ and Teager energy operator^6^, followed by a correction algorithm. Further, the removal of the CB artifact is performed based on the Optimal Basis Set (OBS) method^7^. This step of the truncation of prior EEG data before CB correction has yielded a good quality of CB artifact removal.

1. **Supervised use of functionalities of Harvard Automated Processing Pipeline for Electroencephalography (HAPPE):**

In addition to the above steps, we have also employed the HAPPE toolbox^8^ for further ensuring the quality of conventional EEG artifact removal from the scanner and CB artifact corrected datasets. The steps adopted in HAPPE toolbox are,

1. The scanner and CB artifact removed data were first subjected to the filtering process with 0.1 Hz High pass and 70 Hz low pass filtering, and all the EEG channels were selected for further analysis.
2. The electrical (line) noise was removed using the Cleanline plugin^9^ of EEGLAB.
3. The functionality of HAPPE was utilized next to identify and remove the contaminated channels. HAPPE identifies the contaminated channels by evaluating the normed joint probability of average log power across all the channels and rejecting the channels whose joint probability is more than three standard deviations.
4. Wavelet enhanced ICA (W-ICA) approach was implemented subsequently to correct for EEG artifact while retaining the entire length of the data file. The W-ICA approach removes ocular and muscle-related artifacts and also improves the decomposition of later performed ICA, which eventually rejects artifact components.
5. Next, independent components (ICs) with the extended infomax independent component analysis (ICA) were computed, and the MARA plugin^10,11^ of EEGLAB was employed for automatic component rejection. MARA evaluates each component on six features and eventually assigns a probability of artifact contamination to that component. Further, HAPPE's pipeline automatically rejected any components with artifact probabilities higher than 0.5.
6. Subsequently, segmentation of data based on the markers, rejection of segments, interpolation of removed channels, were carried out.
7. Finally, the processing report about the quality of data was generated. This report for all volunteers has been tabulated in Supplementary Table 1, which establishes the quality of final EEG datasets.
8. **The power spectrum of the final artifact removed EEG data:**

To validate the removal of both MR and conventional artifacts from the raw EEG data, we subjected the final artifact removed EEG data (CSD referenced) to the estimation of the power spectrum between 0.2 Hz to 50 Hz frequency range. The median power spectrum plots of the final artifact removed EEG data for channels F3, F4, F7, F8, Pz, Oz, and POz is shown in Supplementary Fig. 2. While comparing Supplementary Fig. 1 with Supplementary Fig. 2, it is evident that,

1. The MR gradient artifact peaks observed at the 15, 30, 45 Hz (i.e., multiples of no. of slices/TR) range in raw data have been appropriately cleaned.
2. The median spectral power of artifact removed EEG data reveals parietal and occipital alpha and beta bands.

**References**:

1. Allen, P. J., Josephs, O. & Turner, R. A method for removing imaging artifact from continuous EEG recorded during functional MRI. *NeuroImage* **12**, 230–239 (2000).

2. Allen, P. J., Polizzi, G., Krakow, K., Fish, D. R. & Lemieux, L. Identification of EEG events in the MR scanner: The problem of pulse artifact and a method for its subtraction. *Neuroimage* **8**, 229–239 (1998).

3. Ritter, P., Becker, R., Freyer, F. & Villringer, A. EEG quality: The image acquisition artefact. *EEG - fMRI Physiol. Basis, Tech. Appl.* 153–171 (2010). doi:10.1007/978-3-540-87919-0_9

4. Ritter, P., Becker, R., Graefe, C. & Villringer, A. Evaluating gradient artifact correction of EEG data acquired simultaneously with fMRI. *Magn. Reson. Imaging* **25**, 923–932 (2007).

5. Niazy, R. K., Beckmann, C. F., Iannetti, G. D., Brady, J. M. & Smith, S. M. Removal of FMRI environment artifacts from EEG data using optimal basis sets. *Neuroimage* **28**, 720–737 (2005).

6. Christov, I. I. Real time electrocardiogram QRS detection using combined adaptive threshold. *Biomed. Eng. Online* **3**, 1–9 (2004).

7. Kim, K. H., Yoon, H. W. & Park, H. W. Improved ballistocardiac artifact removal from the electroencephalogram recorded in fMRI. *J. Neurosci. Methods* **135**, 193–203 (2004).

8. Gabard-Durnam, L. J., Leal, A. S. M., Wilkinson, C. L. & Levin, A. R. The harvard automated processing pipeline for electroencephalography (HAPPE): Standardized processing software for developmental and high-artifact data. *Front. Neurosci.* **12**, 1–24 (2018).

9. Mullen, T. CleanLine EEGLAB Plugin. San Diego, CA: Neuroimaging Informatics Toolsand Resources Clearinghouse (NITRC). (2012).

10. Winkler, I., Haufe, S. & Tangermann, M. Automatic Classification of Artifactual ICA-Components for Artifact Removal in EEG Signals. *Behav. Brain Funct.* **7**, 1–15 (2011).

11. Winkler, I. *et al.* Robust artifactual independent component classification for BCI practitioners. *J. Neural Eng.* **11**, (2014).

**SUPPLEMENTARY TABLE LEGEND**

**Supplementary Table 1:** HAPPE preprocessing report generated for all volunteers' EEG datasets.

**SUPPLEMENTARY TABLE**

**Supplementary Table 1:** HAPPE preprocessing report generated for all volunteers' EEG datasets.

| Volunteers' File Number | Volunteers' File Length In Secs | Number Of Channels User Selected (excluding ECG) | Number of Segments Post Segment Rejection | Number Of Good Channels Selected (excluding ECG) | Percent Of Good Channel Selected (excluding ECG) | Interpolated Channel  IDs | Number Of ICs Rejected | Percent Of ICs Rejected | Percent Variance Kept Of  Post Waveleted Data | Median Artifact Probability  Of Kept ICs | Mean Artifact Probability  Of Kept ICs | Range Artifact Probability  Of Kept ICs | Min Artifact Probability  Of Kept ICs | Max Artifact Probability  Of Kept ICs |
| --- | --- | --- | --- | --- | --- | --- | --- | --- | --- | --- | --- | --- | --- | --- |
|  |  |  |  |  |  |  |  |  |  |  |  |  |  |  |
|  |  |  |  |  |  |  |  |  |  |  |  |  |  |  |
|  |  |  |  |  |  |  |  |  |  |  |  |  |  |  |
|  |  |  |  |  |  |  |  |  |  |  |  |  |  |  |
| Volunteer_1 | 410.0 | 31 | 1 | 31 | 96.875 | - | 2 | 6.5 | 96.4 | 0.02 | 0.07 | 0.47 | 0.00185 | 0.47 |
| Volunteer_2 | 410.0 | 31 | 1 | 29 | 90.625 | Cz FC1 | 6 | 20.7 | 89.1 | 0.04 | 0.09 | 0.41 | 0.00258 | 0.42 |
| Volunteer_3 | 410.0 | 31 | 1 | 31 | 96.875 | - | 5 | 16.1 | 93.3 | 0.14 | 0.16 | 0.43 | 0.00033 | 0.43 |
| Volunteer_4 | 410.0 | 31 | 1 | 29 | 90.625 | Cz FC2 | 5 | 17.2 | 96.3 | 0.08 | 0.12 | 0.38 | 0.00026 | 0.38 |
| Volunteer_5 | 410.0 | 31 | 1 | 31 | 96.875 | - | 2 | 6.5 | 98.2 | 0.06 | 0.10 | 0.27 | 0.00024 | 0.27 |
| Volunteer_6 | 410.0 | 31 | 1 | 31 | 96.875 | - | 0 | 0.0 | 100.0 | 0.03 | 0.07 | 0.43 | 0.00019 | 0.43 |
| Volunteer_7 | 410.0 | 31 | 1 | 31 | 96.875 | - | 2 | 6.5 | 97.9 | 0.03 | 0.09 | 0.43 | 0.00026 | 0.43 |
| Volunteer_8 | 410.0 | 31 | 1 | 31 | 96.875 | - | 3 | 9.7 | 96.7 | 0.05 | 0.12 | 0.49 | 0.00014 | 0.49 |
| Volunteer_9 | 410.0 | 31 | 1 | 31 | 96.875 | - | 2 | 6.5 | 96.8 | 0.02 | 0.10 | 0.47 | 0.00088 | 0.47 |
| Volunteer_10 | 410.0 | 31 | 1 | 31 | 96.875 | - | 3 | 9.7 | 97.8 | 0.03 | 0.05 | 0.24 | 0.00102 | 0.25 |
| Volunteer_11 | 410.0 | 31 | 1 | 30 | 93.75 | Cz | 4 | 13.3 | 96.3 | 0.14 | 0.13 | 0.42 | 0.00161 | 0.42 |
| Volunteer_12 | 410.0 | 31 | 1 | 30 | 93.75 | Cz | 2 | 6.7 | 99.0 | 0.04 | 0.11 | 0.49 | 0.00055 | 0.49 |
| Volunteer_13 | 410.0 | 31 | 1 | 31 | 96.875 | - | 3 | 9.7 | 96.6 | 0.08 | 0.12 | 0.38 | 0.00031 | 0.38 |
| Volunteer_14 | 410.0 | 31 | 1 | 31 | 96.875 | - | 2 | 6.5 | 98.4 | 0.02 | 0.07 | 0.50 | 0.00029 | 0.50 |
| Volunteer_15 | 410.0 | 31 | 1 | 29 | 90.625 | FC1 Fz | 0 | 0.0 | 100.0 | 0.02 | 0.05 | 0.34 | 0.00015 | 0.34 |
| Volunteer_16 | 410.0 | 31 | 1 | 30 | 93.75 | Cz | 5 | 16.7 | 95.1 | 0.09 | 0.10 | 0.42 | 0.00027 | 0.42 |
| Volunteer_17 | 410.0 | 31 | 1 | 31 | 96.875 | - | 3 | 9.7 | 97.1 | 0.04 | 0.06 | 0.37 | 0.00076 | 0.37 |
| Volunteer_18 | 410.0 | 31 | 1 | 30 | 93.75 | Cz | 3 | 10.0 | 93.2 | 0.03 | 0.05 | 0.21 | 0.00067 | 0.21 |
| Volunteer_19 | 410.0 | 31 | 1 | 31 | 96.875 | - | 1 | 3.2 | 99.6 | 0.02 | 0.07 | 0.43 | 0.00037 | 0.43 |
| Volunteer_20 | 410.0 | 31 | 1 | 30 | 93.75 | Fz | 0 | 0.0 | 100.0 | 0.04 | 0.07 | 0.31 | 0.00054 | 0.31 |
| Volunteer_21 | 410.0 | 31 | 1 | 31 | 96.875 | - | 8 | 25.8 | 92.6 | 0.19 | 0.19 | 0.47 | 0.00149 | 0.48 |
| Volunteer_22 | 410.0 | 31 | 1 | 31 | 96.875 | - | 5 | 16.1 | 92.6 | 0.16 | 0.17 | 0.44 | 0.00648 | 0.44 |
| Volunteer_23 | 410.0 | 31 | 1 | 29 | 90.625 | Fz TP9 | 2 | 6.9 | 99.0 | 0.04 | 0.08 | 0.48 | 0.00010 | 0.48 |
| Volunteer_24 | 410.0 | 31 | 1 | 30 | 93.75 | Cz | 2 | 6.7 | 99.2 | 0.03 | 0.07 | 0.31 | 0.00002 | 0.31 |
| Volunteer_25 | 410.0 | 31 | 1 | 30 | 93.75 | TP9 | 12 | 40.0 | 72.4 | 0.11 | 0.15 | 0.49 | 0.00137 | 0.49 |
| Volunteer_26 | 410.0 | 31 | 1 | 31 | 96.875 | - | 2 | 6.5 | 98.2 | 0.05 | 0.12 | 0.48 | 0.00051 | 0.48 |
| Volunteer_27 | 410.0 | 31 | 1 | 31 | 96.875 | - | 2 | 6.5 | 99.3 | 0.02 | 0.04 | 0.21 | 0.00008 | 0.21 |
| Volunteer_28 | 410.0 | 31 | 1 | 30 | 93.75 | Fz | 2 | 6.7 | 98.4 | 0.02 | 0.06 | 0.30 | 0.00016 | 0.30 |
| Volunteer_29 | 410.0 | 31 | 1 | 31 | 96.875 | - | 0 | 0.0 | 100.0 | 0.01 | 0.06 | 0.36 | 0.00010 | 0.36 |
| Volunteer_30 | 410.0 | 31 | 1 | 31 | 96.875 | - | 0 | 0.0 | 100.0 | 0.01 | 0.03 | 0.19 | 0.00003 | 0.19 |
| Volunteer_31 | 410.0 | 31 | 1 | 31 | 96.875 | - | 7 | 22.6 | 92.5 | 0.07 | 0.12 | 0.48 | 0.00029 | 0.48 |
| Volunteer_32 | 410.0 | 31 | 1 | 31 | 96.875 | - | 3 | 9.7 | 97.2 | 0.09 | 0.14 | 0.49 | 0.00097 | 0.49 |
| Volunteer_33 | 410.0 | 31 | 1 | 31 | 96.875 | - | 6 | 19.4 | 87.7 | 0.06 | 0.13 | 0.47 | 0.00351 | 0.48 |
| Volunteer_34 | 410.0 | 31 | 1 | 30 | 93.75 | Cz | 5 | 16.7 | 93.5 | 0.08 | 0.13 | 0.40 | 0.00216 | 0.41 |
| Volunteer_35 | 410.0 | 31 | 1 | 30 | 93.75 | Cz | 7 | 23.3 | 90.5 | 0.10 | 0.18 | 0.47 | 0.00147 | 0.47 |
| Volunteer_36 | 410.0 | 31 | 1 | 30 | 93.75 | Fz | 1 | 3.3 | 99.8 | 0.04 | 0.10 | 0.40 | 0.00030 | 0.40 |
| Volunteer_37 | 410.0 | 31 | 1 | 31 | 96.875 | - | 4 | 12.9 | 96.3 | 0.05 | 0.10 | 0.41 | 0.00022 | 0.41 |
| Volunteer_38 | 410.0 | 31 | 1 | 30 | 93.75 | Cz | 6 | 20.0 | 84.1 | 0.06 | 0.15 | 0.48 | 0.00049 | 0.48 |
| Volunteer_39 | 410.0 | 31 | 1 | 31 | 96.875 | - | 2 | 6.5 | 99.2 | 0.02 | 0.09 | 0.43 | 0.00036 | 0.43 |

**SUPPLEMENTARY FIGURE LEGENDS**

**Supplementary Fig. 1:** Median power spectrum (0.2 to 50 Hz) of raw artifact laden data for channels F3, F4, F7, F8, Pz, Oz, and POz.

**Supplementary Fig. 2**: Median power spectrum (0.2 to 50 Hz) of final artifact removed EEG data (CSD referenced) for channels F3, F4, F7, F8, Pz, Oz, and POz.

**SUPPLEMENTARY FIGURES**

**
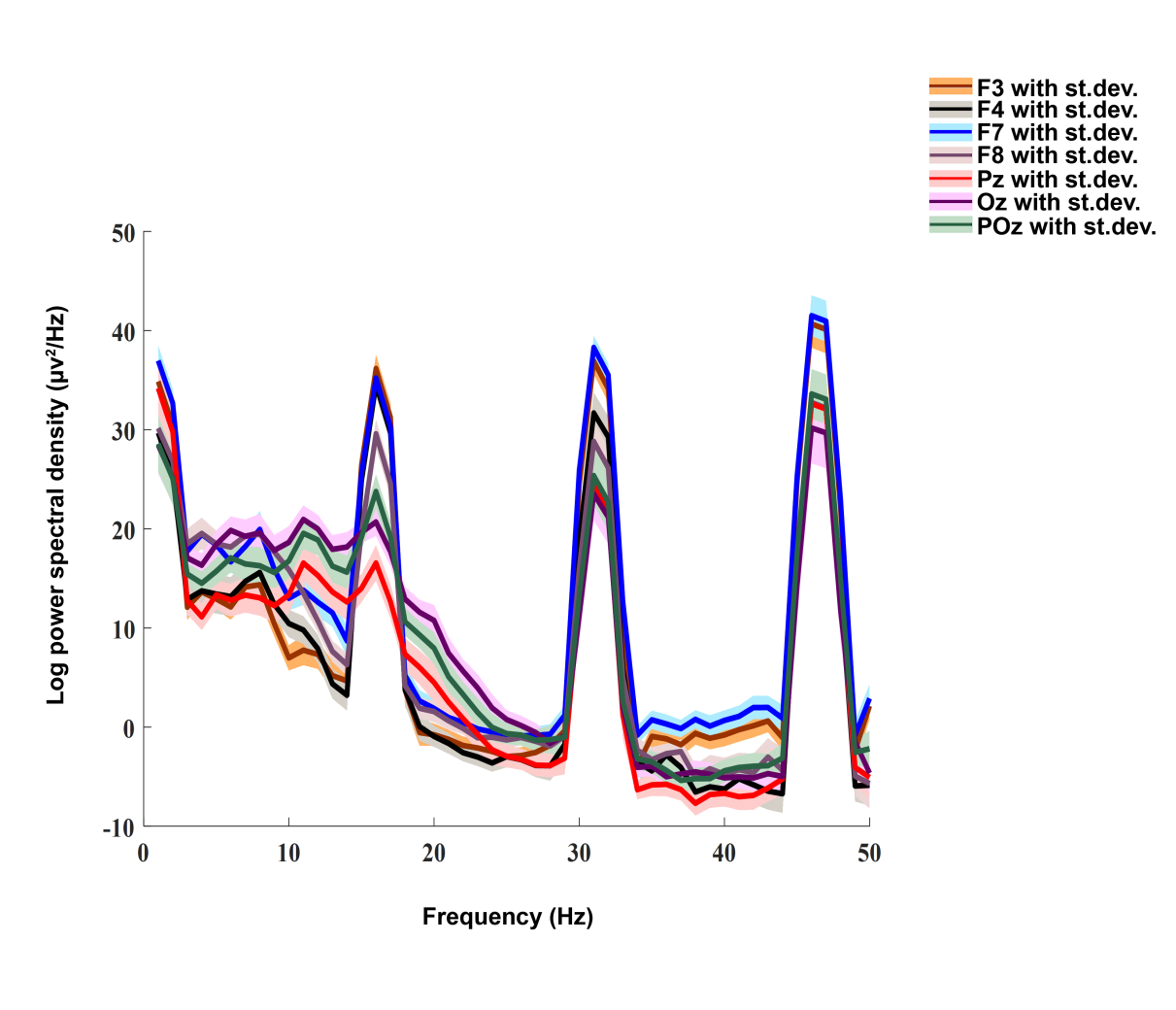
**

**Supplementary Fig. 1:** Median power spectrum (0.2 to 50 Hz) of raw artifact laden data for channels F3, F4, F7, F8, Pz, Oz, and POz.


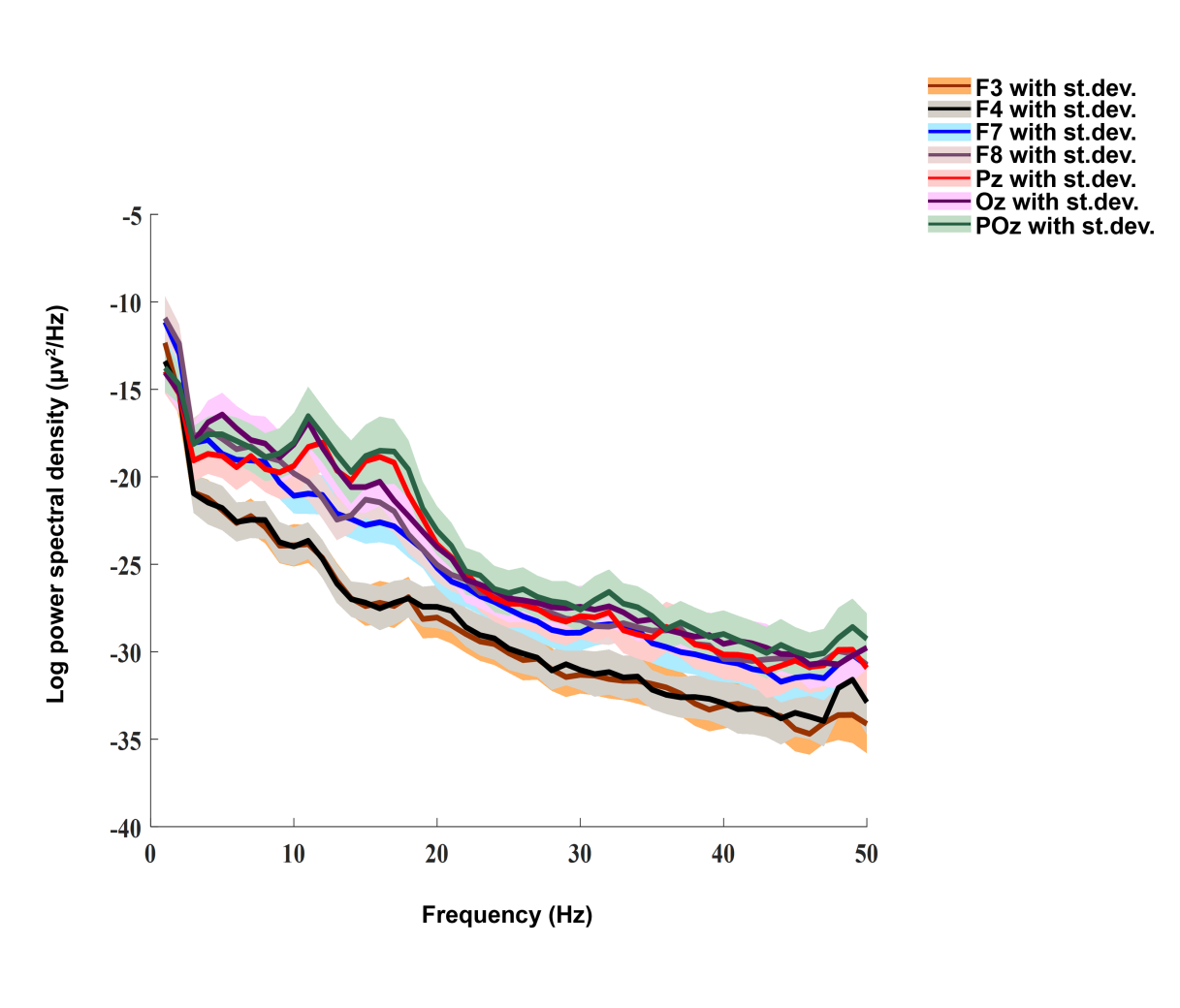


**Supplementary Fig. 2**: Median power spectrum (0.2 to 50 Hz) of final artifact removed EEG data (CSD referenced) for channels F3, F4, F7, F8, Pz, Oz, and POz.
